# Constructing a lower-bound estimate of the global number of insect species on a hyperdiverse empirical foundation

**DOI:** 10.1073/pnas.2524283123

**Published:** 2026-06-29

**Authors:** Robert K. Colwell, Laura Melissa Guzman, Dirk Steinke, Anne Chao, Daniel H. Janzen, Winnie Hallwachs, Austin Baker, José L. Fernández-Triana, Paul D. N. Hebert, Frank Joyce, Robert Puschendorf, Donald L. J. Quicke, Rodolphe Rougerie, M. Alex Smith, Nelson Zamora, Michael J. Sharkey

**Affiliations:** ^a^ Department of Ecology and Evolutionary Biology, University of Connecticut, Storrs, CT 06269-3043; ^b^Entomology Section, University of Colorado Museum of Natural History, Boulder, CO 80309-0001; ^c^ Department of Entomology, Cornell University, Ithaca, NY 14853-2601; ^d^ Centre for Biodiversity Genomics, Department of Integrative Biology, University of Guelph, Guelph, ON N1G2W1, Canada; ^e^ Institute of Statistics, National Tsing Hua University, Hsin-Chu 30043, Taiwan; ^f^ Department of Biology, University of Pennsylvania, Philadelphia, PA 19104-6018; ^g^ Department of Entomology, Natural History Museum of Los Angeles County, Los Angeles, CA 90007; ^h^Canadian National Collection of Insects, Ottawa, ON K1A 0C6, Canada; ^i^Monteverde Institute, Monteverde, Puntarenas 60109, Costa Rica; ^j^ School of Biological and Marine Sciences, University of Plymouth, Plymouth PL4 8AA, United Kingdom; ^k^ Center of Excellence in Integrative Insect Ecology, Department of Biology, Faculty of Science, Chulalongkorn University, Bangkok 10330, Thailand; ^l^Department of Origins and Evolution, Institut de Systématique, Évolution, Biodiversité, Muséum National d’Histoire Naturelle, CNRS, École Pratique des Hautes Études – Université Paris Sciences & Lettres, Sorbonne Université, Université des Antilles, Paris F-75005, France; ^m^ Department of Integrative Biology, University of Guelph, Guelph, ON N1G2W1, Canada; ^n^ Escuela de Ingeniería Forestal, Instituto Tecnológico de Costa Rica, Cartago 159-7050, Costa Rica; ^o^The Hymenoptera Institute, Lexington, KY 40502

**Keywords:** DNA barcodes, species richness, biodiversity, hyperdiverse taxa, Microgastrinae

## Abstract

Estimating the number of insect species on Earth is a daunting challenge. The current consensus estimate—about six million species—is likely far too low, as we will show. Our estimate of the global number of insect species rests on a sample of more than 1,600,000 DNA-barcoded insect specimens representing 53,945 species from 15 “core” Malaise traps deployed in dry forest, cloud forest, and rainforest ecosystems of the Área de Conservación Guanacaste (ACG) in Costa Rica. Even this massive sample fails to reveal the full extent of ACG insect species richness. To estimate total ACG insect richness, we adjust the observed count of insect species by an “undersampling ratio,” computed for a hyperdiverse subfamily of parasitoid wasps (Braconidae: Microgastrinae). The ratio compares microgastrine richness from the core Malaise traps to a lower-bound estimate of true microgastrine richness—including undetected species—based on 21,669 specimens from three sources: the 15 core Malaise traps, 15 “peripheral” Malaise traps spanning all three ecosystems, and 11,373 DNA-barcoded specimens reared from some 1,500 species of microgastrine-parasitized caterpillars (Lepidoptera). To estimate global insect richness, we apply Earth/ACG ratios for tree species and several animal taxa to upscale our estimate of ACG insect richness (nearly 333,000 species). Adopting conservative assumptions, we reach an estimate of 14 to 20 million insect species on Earth, depending on the upscaling group—two to three times the current consensus estimates. Upscaling instead from a point estimate of ACG richness with a wide CI, global estimates reach nearly 30 million species.

## Significance

For more than 40 y, entomologists have attempted to estimate the number of insect species on Earth, with the current consensus—the figure most experts accept—at about six million. Using genetic information (DNA barcodes) for 1.6 million individual tropical insects, a deep census of a highly diverse group of parasitoid wasps, and powerful statistical strategies, we conservatively estimate that the true number of insect species is at least 14 to 20 million—two to three times higher than current estimates. Already known to be the most diverse group of animals, a doubling or tripling of estimated insect diversity has profound implications for our understanding of the scale, richness, and future of biodiversity on Earth.

## Estimating Global Insect Species Richness.

The number of bird species known to science increases by single digits each year, and each discovery typically merits a news story and a unique Linnaean name (1). The discovery of new species of insects—especially tiny tropical ones—gets little public notice, and most of them remain unnamed. Instead, although morphological descriptions and discriminations are certainly still widely used (2), taxonomist specialists of hyperdiverse insect groups increasingly assign each putative species a DNA Barcode Index Number (a “BIN”)—as a proxy for a species name (3–5). In tropical forests, intensive sampling for hyperdiverse groups routinely reveals one rare species after another, only slowly approaching an asymptote of species richness (6, 7). In this study, we apply DNA barcoding and statistical richness estimation (including undetected species), leveraging a classic ratio approach to project a global estimate of insect richness.

Previous estimates of global insect species richness vary widely. The most provocative was Terry Erwin’s (8) back-of-the-envelope guess of 30 million species of terrestrial arthropods, which stimulated a long series of reconsiderations and critiques [reviewed by Stork (9) and García‐Robledo et al. (10)]. Among recent estimates of global insect richness, however, most are around six million (9, 11–13), with Li and Wiens (14) an outlier at 21 million species. Beetles (Coleoptera) have been believed, by long tradition, to be the most diverse order of insects (8, 13, 15–17). However, richness estimates and the claim that beetles are the most diverse order have been challenged by discoveries that DNA barcoding reveals an immense, undescribed species richness of tiny flies (Diptera: Nematocera and Phoridae) (18–20) and parasitoid wasps (Hymenoptera: Apocrita) (9, 21–26).

Here, based upon an extensive DNA barcode dataset for a hyperdiverse tropical insect fauna and the application of rigorous statistical methods, we propose a substantial increase in the lower-bound estimate for global species richness of insects. We calibrate the estimate through deep sampling of a subfamily of parasitoid wasps from a small, but climatically and microclimatically diverse, multiecosystem region in Costa Rica.

## Strategy

### Step 1: The Foundation.

**Fig. 1.**
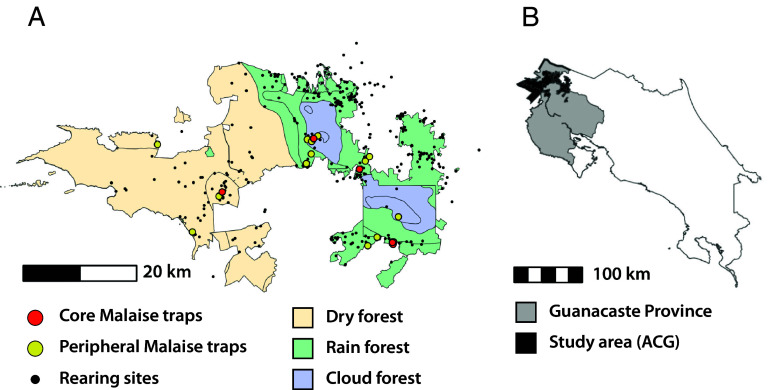
(*A*) Distribution of “core” and “peripheral” Malaise traps and capture sites for caterpillars yielding Microgastrinae by rearing. Points were jittered to reveal overlapping points. Each core Malaise point (red) represents three nearby traps. Each peripheral Malaise point (yellow) represents one or two nearby traps. Points outside current ACG boundaries lay within ACG ecosystems at the time of capture. (*B*) Map showing the study area (black) within ACG, in the Province of Guanacaste, Costa Rica (gray).

The foundation for our estimate of the global number of insect species is a massive sample of 1,633,855 insect specimens from 15 “core” Malaise traps operated for a combined 69 trap-years (a trap-year is one trap operating for 1 y). The 15 core traps were deployed along a 100-km transect from Pacific lowland dry forest (300 m) to mid-elevation cloud forest (1,300 m) and across the Continental Divide into lowland Caribbean rainforest (575 m) in Costa Rica’s Área de Conservación Guanacaste (ACG) (23, 27–31). All insects from these samples were barcoded, regardless of taxon. Core Malaise traps are indicated by red dots in Fig. 1.

### Step 2: Estimating a Benchmark: Microgastrinae Richness.

Even such a large sample fails to reveal the full extent of ACG insect species richness. To estimate full ACG insect richness, in the next step we adjust the observed count of insect species from core Malaise traps by an “undersampling ratio,” estimated by comparing the observed core Malaise richness of a single, hyperdiverse, subfamily of parasitoid wasps (Braconidae: Microgastrinae) to a statistical lower-bound estimate (with confidence limits) of true microgastrine richness—including undetected species. We base this comprehensive estimate on microgastrine specimens from three intensively sampled sources: the 15 core Malaise traps, an additional 15 “peripheral” Malaise traps spanning all three ecosystems (yellow dots in Fig. 1), and 11,373 DNA-barcoded specimens reared from some 1,500 species of microgastrine-parasitized caterpillars, sampled by hand from locations throughout the transect (black dots in Fig. 1). Only microgastrines were comprehensively removed from the catch in the peripheral Malaise traps and DNA barcoded, so no other taxon was available to be used in this way.

To combine ACG Microgastrinae data from the core and peripheral Malaise traps with microgastrine data from captive rearing of adult wasps, we apply Chao’s “one-step sample coverage richness estimator” *Nhat–*1 (32, 33) in the CARE*-*1 package (34). Based on simulation studies (32, 33), this lower-bound estimator is typically accurate [in the sense of ISO 5725-1:2023 (35)], (i.e., it exhibits low bias and low variance). Applied to these three sets of microgastrine data, *Nhat–*1 yields an estimate of the statistical lower bound of true species richness for Microgastrinae in ACG—our preferred benchmark for true ACG richness. The CARE-1 package also yields an approximate point estimate (*N-hat*) of true ACG Microgastrinae richness, with a wide CI, which we report for comparison.

### Step 3: Estimating the Undersampling Ratio for Core Microgastrinae.

By comparing the observed (core) microgastrine richness from the 15 core Malaise traps to the benchmark estimate of true ACG microgastrine richness from Step 2, we estimate an approximate level of microgastrine undersampling by the core Malaise traps—with their much smaller sample size and single sampling method, compared to the pooled and extrapolated data for Microgastrinae.

### Step 4: Estimating the True Species Richness of ACG Insecta.

Assuming the same level of undersampling applies to the core Malaise richness for all insects, we adjust that number proportionally upward, to secure a lower-bound estimate of the total species richness of ACG insects.

### Step 5: Estimating Global Insect Richness.

Treating the ratio between published estimates of the number of tree species on Earth and an expert estimate of the number of tree species in ACG as an “upscaling factor,” we project our lower-bound estimate of ACG insect richness to global insect richness, with CI. For comparison, we repeat this step with estimated numbers of global and ACG mammals, amphibians, and Saturniidae moths.

We reference these numbered steps, as appropriate, in *Results* and the *Discussion* sections.

## Results

### Overview.

Through a sequence of linked steps, reported in detail below, we reached a set of four alternative lower-bound estimates of the number of insect species on Earth. We first estimated the true richness of ACG insects (including undetected species) by scaling up from a deep sample of Microgastrinae—a hyperdiverse subfamily of braconid wasps—treating BINs as species. We applied Earth/ACG ratios for tree species and several animal taxa to upscale our estimate of ACG insect richness to the global scale.

### Step 1: The Foundation.

The 15 core Malaise traps (69 trap-years) produced a total of 1,633,855 insect specimens, all subsequently barcoded, yielding a total of 53,945 species (BINs).

### Step 2: Estimating a Benchmark: Microgastrinae Richness.

**Fig. 2.**
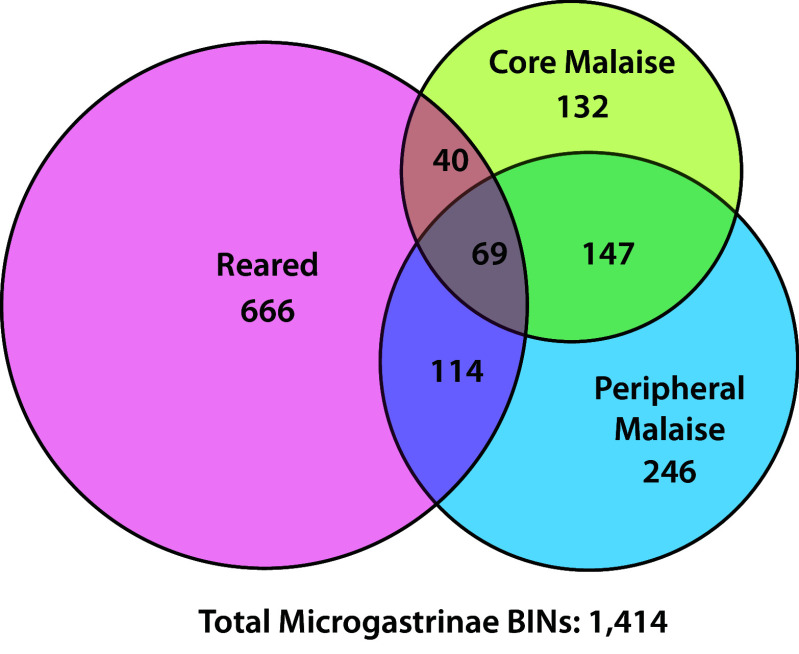
Quantitative Venn diagram showing the number of Microgastrinae species (BINs) exclusive to reared specimens, core Malaise, and peripheral Malaise trap samples and the number shared between pairs of sample sets and in common to all three sample sets.

As of December 2024, the 15 core Malaise traps had yielded 3,781 microgastrine specimens, all subsequently barcoded, representing 388 species (BINs); the 15 peripheral Malaise traps had produced 6,515 microgastrine specimens of 576 species; and the 11,373 microgastrine specimens from captive rearing of host caterpillars represented 889 species. Fig. 2 shows the number of Microgastrinae species exclusive to reared specimens, core Malaise, and peripheral Malaise trap samples, the number shared between pairs of sample sets, and the number common to all three sample sets, totaling 1,414 microgastrine species. The three sets of samples captured relatively distinct faunas, with three-quarters of the species exclusive to one or another of the three sample sets, although larger samples collected over additional years would likely reduce this proportion. *SI Appendix*, Fig. S1 illustrates, for additional insect ACG taxa, the striking degree to which rearing and Malaise trapping reveal different community components at the same sampling site (ACG).

Of the 1,414 observed species, 403 (29%) were represented by singletons (one specimen only). Clearly, despite the very large, pooled sample (21,669 specimens), ACG Microgastrinae remain undersampled (7). To estimate the true number of ACG Microgastrinae species, including those not detected in any of the three samples, we applied Chao’s “one-step” sample coverage richness estimator, *Nhat–*1, in the CARE-1 package (34). In epidemiology, this approach is used to estimate the true size of target populations when three or more methods of ascertainment have been used to identify affected individuals. The only data required are the seven incidence values (counts) in Fig. 2. Abundance data are not needed.

The CARE-1 method yields an accurate [in the sense of ISO 5725-1:2023 (35)], lower-bound estimate, *Nhat*-1, and an approximate point estimate, *Nhat*. Each has its own 95% CI. The lower-bound estimate of ACG Microgastrinae richness (with 95% CI) was 2,394 [2,221; 2,604] species (where [*n*; *m*] is the 95% CI), with an estimated sampling completeness [observed richness divided by estimated true richness (36)] of 0.59 [0.54; 0.64]. In contrast, the point estimate of ACG Microgastrinae richness was 3,405 [2,493; 5,088] species, with an estimated sampling completeness of only 0.42 [0.28; 0.57]. The uncertainty of the point estimate is evident from its wide CI for richness—76% of the estimate, compared to just 16% of the estimate of the lower-bound species richness estimate. In line with other elements of our conservative approach to estimating global insect richness, we focus, henceforth, on the statistically more accurate estimate of the lower-bound of species richness. For completeness, results based on the more approximate point estimate are reported in Dataset S1.

### Step 3. Estimating the Undersampling Ratio for Core Microgastrinae.

The 1,633,855 insect specimens from the core Malaise samples that yielded a BIN-compliant sequence represented a total of 53,945 species (BINs), among which 388 species were Microgastrinae. The lower-bound estimate of ACG Microgastrinae richness, for all three sampling methods pooled (including core Malaise) was 2,394 [2,221; 2,604] as detailed above. Thus, the estimated undersampling ratio, *UR*, for core Malaise Microgastrinae was *UR* = 388/2,394 = 0.162 [0.148; 0.176].

### Step 4. Estimating the True Species Richness of ACG Insecta.

#### Hierarchical taxon-ratio projection.

The principal assumption behind taxon-ratio estimates of true species richness (the number of species observed plus the number present but not detected) is that a lower taxon (e.g., a subfamily, such as Microgastrinae) that has been targeted for especially intensive sampling can be treated as an indicator of the degree of undersampling of a higher taxon (such as Insecta)—taxonomically more inclusive but less intensively sampled (37). But the projection must, of course, be based on a subset of the lower taxon samples that arise from the same samples as the higher taxon—in our case, from core Malaise trap samples. Mathematically, the taxon-ratio assumption is that the undersampling ratio of the subtaxon, *UR* = *S*_obs_/*S*_est_, approximates the undersampling ratio (sampling incompleteness) of the higher taxon, *T*_obs_/*T*_est_, where the subscript *obs* is the empirical tally of species and the subscript *est* indicates the estimated true richness, including species present, but not detected. The estimate of the true richness of the higher taxon, *T*_est_, is unknown. Solving for it yields

$$T_{\text{est}}=T_{\text{obs}}\times [S_{\text{est}}/S_{\text{obs}}]=T_{\text{obs}}/UR.$$

#### Estimating ACG insect species richness.

The core Malaise traps yielded 53,945 insect species (BINs). Following the logic and assumptions of taxon-ratio projection, the estimated true species richness of Insecta in ACG, including species present but undetected, is

$$\begin{array}{c} T_{\text{est}}=T_{\text{obs}}/UR=53,945/(388/2,394)\;[0.148;\;0.176;]\\ \;=332,846\;[306,847;\;364,963]. \end{array}$$

### Step 5. Estimating Global Insect Richness.

To estimate the global species richness of insects, we projected the estimated species richness of ACG insects to a global scale, initially using the ratio between the estimated global tree richness and estimated ACG tree richness as a guide. Trees are our preferred upscaling reference group, as explained in the Discussion. To estimate insect richness for the Earth we assumed that

$$\frac{\text{Insecta}(\text{ACG})}{\text{Insecta}(\text{Earth})}=\frac{\text{Trees}(\text{ACG})}{\text{Trees}(\text{Earth})}.$$

Solving for Insecta(Earth), we get

$$\text{Insecta}(\text{Earth})=\text{Insecta}(\text{ACG})\times \left[\frac{\text{Trees}(\text{Earth})}{\text{Trees}(\text{ACG})}\right].$$

We refer to the ratio Trees(Earth)/Trees(ACG) as a richness “upscaling factor.”

The number of tree species in ACG was estimated by coauthor Nelson Zamora (38, 39), Associate Curator of the Herbarium of the Museo Nacional de Costa Rica and leading authority on Costa Rican trees. Zamora estimated the ACG tree flora at 1,200 to 1,500 species. Statistical estimates of the number of tree species on Earth (73,274 species, corrected for probable false uniques; 89,147 species uncorrected) are from Cazzolla Gatti et al. (40), with an independent count (60,065 species) of described (named) tree species from Beech et al. (41).

Cazzolla Gatti et al. (40) estimated global tree richness based on incidence (presence) data for gridded maps. For their more conservative estimate (73,274 tree species), they applied the method of Chiu and Chao (42), which adjusts for possible spurious “uniques”—species recorded in only one grid cell (37), a substantial proportion of which are likely to be synonyms, misspellings, or misidentifications (43). For their alternative estimate (89,147 tree species), Cazzolla Gatti et al. (40) assumed that all uniques are legitimate.

**Fig. 3.**
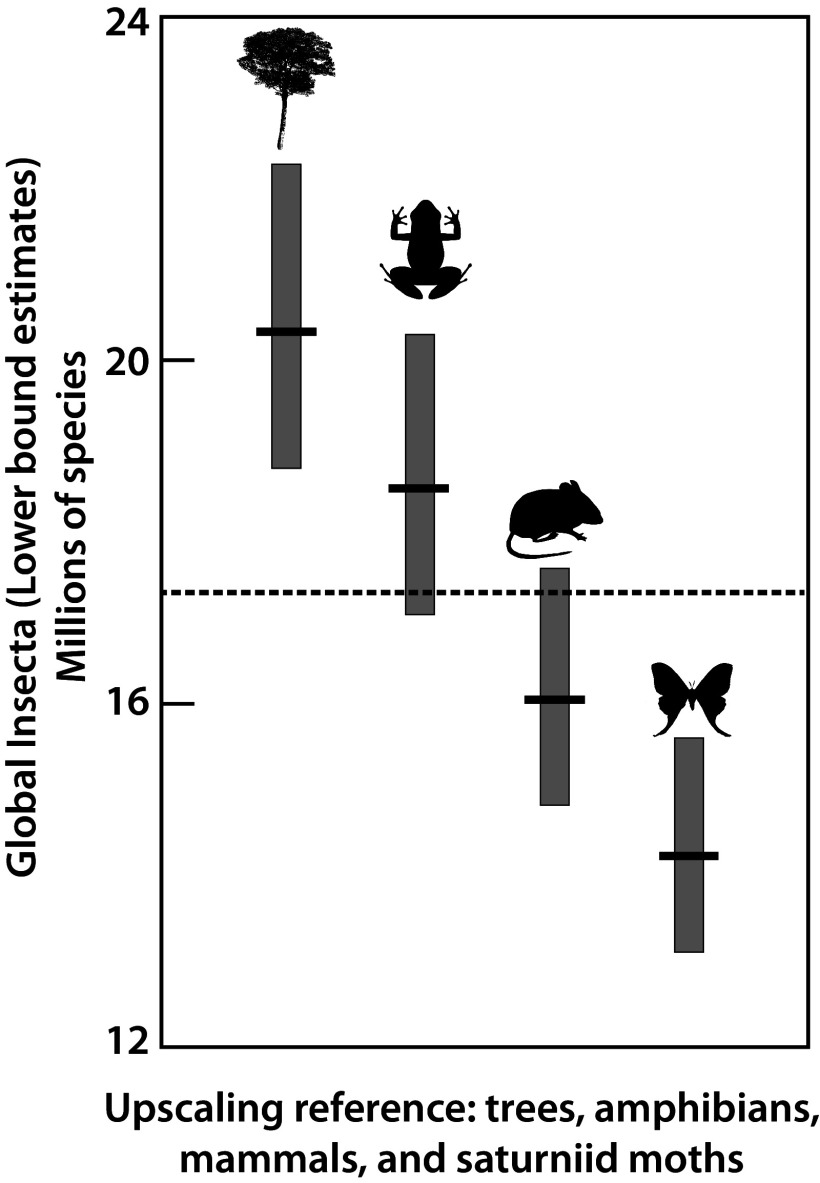
Estimates of global insect species richness, upscaled from estimated ACG insect species richness using ratios of global to ACG richness (richness scaling factors) for four alternative reference groups (Dataset S1). Horizontal black bars are expectations; gray columns are 95% confidence bands. The dashed horizontal line is the mean of the four expectations.

With a lower and upper estimate of ACG tree richness (1,200 to 1,500), two estimates of global tree richness from Cazzolla Gatti et al. (40), and a third from Beech et al. (41), we have six estimates of the richness upscaling factor Trees(Earth)/Trees(ACG), yielding six estimates of global insect richness are possible, ranging from 13,328,263 to 24,726,852 species. We report these estimates, with CI, in Dataset S1. Conservatively, we choose—as representative—the 20,324,132 species estimate of global insect species ([corrected global trees of Cazzolla Gatti]/[1,200 ACG]), relying on the *Nhat*-1 lower-bound estimate of ACG Microgastrinae richness (Fig. 3). This estimate is similar to the estimate of global insect richness of Li and Wiens (14)—21 million species—but reached by an entirely different approach.

To assess the sensitivity of this estimate to choice of richness upscaling group, we searched diligently for other options—groups for which credible estimates of both ACG and global richness are available. We came up with such estimate pairs for amphibians, mammals, birds, odonates (dragonflies and damselflies), saturniid moths (giant silk moths), sphingid moths (hawkmoths), and butterflies. For each group, we also recorded (or computed) median global geographical range size among species, which ranged widely, from 4,510 km^2^ (amphibians) to 1,290,304 km^2^ (odonates). These data and their sources appear in Dataset S1.

Abundant evidence from empirical studies (e.g., ref. 44) and simulations (45) demonstrates that the rate of spatial turnover in local species composition within a spatial domain [conceptually related to beta diversity (46, 47)] is inversely related to range size: groups with smaller ranges tend to have greater species turnover. Our Earth/ACG richness upscaling factors estimate how many times as rich the Earth’s biota is than ACG’s biota, for each group. A large upscaling factor suggests greater spatial turnover than a small upscaling factor, implying smaller geographical ranges. We found a strong signal of this pattern for the eight candidate groups we considered: Richness upscaling factor is inversely related to median global geographical range size (*R*^2^ = 0.6742, |*r*| = effect size = 0.821; *SI Appendix*, Fig. S2 and Dataset S1).

Upscaling ACG insect richness to global insect richness should, ideally, be done using an upscaling reference group that, at least approximately, corresponds in global median range size to ACG insects. Unfortunately, we have no way of assessing the latter variable directly, given the paucity of data on the most diverse tropical insect taxa, outside of ACG. Nonetheless, there is reason to expect that the great majority of ACG insect species—taking into account hyperdiverse groups like parasitoid wasps and flies—have ranges restricted by habitat, host species, and climate, within the topographically diverse extent of the ACG, and thus the high levels of regional spatial species turnover that are characteristic of topographically diverse regions (27, 48, 49).

To assess this conjecture, we plotted the distance decay of similarity (proportion shared species) among all 30 ACG Malaise traps (core and peripheral) for Microgastrinae wasps—the most intensively sampled taxon in the ACG and thus the least affected by undersampling bias (50). The results (*SI Appendix*, Fig. S3) reveal a marked decline in similarity across short distances, a classic indicator of high species turnover and small ranges (51).

We set an arbitrary, but conservatively high upper limit of 300,000 km^2^ (the size of Italy) for median range size for upscaling reference groups, eliminating birds, odonates, sphingid moths, and butterflies, all of which have median range sizes greater than 800,000 km^2^ (the size of Egypt) (Dataset S1). The four remaining groups—trees, amphibians, mammals, and saturniid moths—yielded a range of global insect richness estimates, from 14.2 to 20.3 million species, with a mean among estimates of 17.3 million insect species (Fig. 3).

*SI Appendix*, Table S1 presents additional results, using the same approach, for ACG and global richness for order Hymenoptera, superfamily Ichneumonoidea, family Braconidae, and subfamily Microgastrinae, anchored in the 1,500-species estimate of ACG tree species richness and the conservative estimate of global tree richness (73,274 species) of Cazzolla Gatti et al. (40).

## Discussion

Any quantitative estimate based on sampling data is only as good as the data it relies on and the assumptions it requires. Here, we examine the principal assumptions that underlie our estimates of global insect richness and predict the probable effects on those estimates in the event of the failure of each assumption.

### Step 1: The Foundation.

Barcoded individual insects from the core Malaise traps are the foundation of our strategy for estimating the global number of insect species. We acknowledge that BINs—DNA BINs (3)—are only an approximation to species. To determine the accuracy of the BIN system, Ratnasingham and Hebert (3) investigated eight large datasets, each with all specimens barcoded, but previously assigned to morphologically determined, named species. Combining results from a bee dataset, three Lepidoptera datasets, two fish datasets, and two bird datasets, BINs correctly matched morphological species delimitations 89.2% of the time, split species (assigned specimens of a single morphological species to two or more BINs) at a rate of 2.7%, merged (lumped) species (assigned specimens of two or more morphological species into a single BIN) at 7.9%, and both merged and split species (some specimens of two or more morphological species were assigned to the same BIN and other specimens of one or more of those same species assigned to different BINs) at 0.3%. (The total percentage—100.1—is off by accumulated rounding errors). More important, for our purposes, the correspondence between the number of morphological species and the number of BINs among the eight datasets in the Ratnasingham and Hebert study (3) was *R*^2^ = 0.999. Because we rely strictly on DNA barcodes to estimate insect richness, concerns about cryptic species (14, 52) are minimized.

In general, BINs slightly underestimate species numbers, for the Ratnasingham and Hebert (3) test datasets. In the Sharkey et al. (53) treatment of more than 400 Costa Rican braconids, BINs were very accurate, with an underestimate of just 1%, as several BINs contained multiple species, based on morphology, host use, and small COI differences. In the Fernandez-Triana et al. (54) revision of the braconid genus *Dolichogenidea*, largely based on specimens from ACG, out of 84 species with compliant barcode sequences, only seven cases revealed either multiple BINs per species or more than one species per BIN. Fernandez-Triana et al. (55) found a similar degree of correspondence (90%) for ACG species in the braconid genus *Alphomelon*. An investigation of the Costa Rican macro-Lepidoptera, based on named and unnamed species in BOLD (March 20, 2022), showed an average 10% underestimate of species numbers based on BINs. Thus, the observed numbers of BINs for ACG Microgastrinae and for ACG Insecta, if anything, may be underestimates of the number of morphologically defined species.

### Step 2: Estimating a Benchmark: Microgastrinae Richness.

Estimating the mean, SD, and sexual difference in antenna length in a moth species, based on a sample of individuals, is a routine task requiring routine assumptions. But assumptions are less obvious when the number to be estimated is a countable (discrete) variable—number of species, in our case—sampled from a highly skewed statistical distribution of individuals, representing species that vary widely in relative abundance. The count of species in any practical sample, even a large one, is inevitably negatively biased—an undercount—to a degree directly (but idiosyncratically) dependent on sample size, especially for species-rich assemblages, such as tropical insects (and tropical trees). Not even the most powerful statistical methods for estimating species richness from samples can deliver an accurate point estimate of true richness when many rare species remain undetected, as in the case of ACG Microgastrinae. Nonetheless, these methods can provide an accurate lower bound (56), with confidence limits.

After decades of failed efforts to discover a parametric distribution that consistently describes the frequency distribution of individuals among species [beginning with Fisher et al. (57)], nonparametric estimators have become the consensus choice for estimating true species richness from sampling data, including species present but undetected in sampling records (58). The tool we rely on here, Chao’s “one-step” sample coverage richness estimator, *Nhat*-1, in the CARE-1 package (32, 34) was developed for epidemiological applications. The example that Chao et al. (34) offer for the three-sample case is estimating the true number of hepatitis A cases during an outbreak in a college in Taiwan, based on three methods of ascertainment: a serum test conducted by the Institute of Preventive Medicine, cases reported by doctors at local hospitals, and records based on questionnaires. It was Alan Turing’s discovery (59) that the frequencies of the rarest classes are the key to estimating the frequency of undetected classes and the basis for estimating sample coverage. In the epidemiological example, sample coverage depends on the frequency of cases detected by only one method of ascertainment, in relation to total cases. In our application, sample coverage depends on the frequency of species captured, exclusively, by each of the three sample sources in relation to shared captures (Fig. 2).

Given our preference for a conservative approach, we estimate the richness of ACG Microgastrinae, with the data in hand, treating the *Nhat*-1 richness estimate as the minimum number of microgastrine species that could potentially be revealed with further sampling by the same methods in the same locations—an accurate estimate of a lower bound. In contrast, setting additional Malaise traps at other locations in ACG (or in the canopy, at any location) or rearing Microgastrinae from caterpillars collected more widely (or from the canopy) would almost certainly increase observed and estimated richness, above and beyond the effect of larger sample size itself. Moreover, the ACG rearing program made no attempt to rear wasps from leaf-mining or stem-boring Lepidoptera, although an unknown number of Malaise-trapped microgastrines may have had these origins. Thus, overall, the assumptions of Step 2 are almost certainly conservative—perhaps very conservative—with respect to our estimate of global insect richness.

Microgastrinae were chosen for two reasons as the benchmark reference for upscaling ACG insect richness to account for undersampling. First, as a proxy for all ACG insects, we needed a hyperdiverse group that could be intensively sampled and barcoded. Microgastrinae was already known to be the most species-rich subfamily of parasitic wasps—themselves rivaled, globally, only by microdiptera for richness (18–20). Second, this braconid subfamily had been the focus of especially intense collecting and barcoding throughout the ACG—the only hyperdiverse taxon for which all specimens were barcoded from peripheral Malaise trap captures. Such comprehensive sampling confirmed that reared Microgastrinae emerging from parasitized caterpillars represented a substantially distinct subfauna from those captured by Malaise traps (Fig. 1).

But how typical are Microgastrinae? What if we had chosen a different ichneumonoid subfamily? To assess this question, we compared reared specimens and core Malaise data for 12 additional ichneumonoid subfamilies (*SI Appendix*, Table S2). Because only Microgastrinae were fully extracted and barcoded from the peripheral Malaise samples, we restricted comparison among subfamilies—including Microgastrinae—to data for reared specimens and core Malaise samples. We tallied three attributes of each subfamily: number of observed species, proportion of species shared between reared specimens and core Malaise samples, and sampling completeness (*S*_obs_/i*Chao*1) (36). Reassuringly, Microgastrinae is by far the most diverse of the 15 subfamilies, but it is not extraordinary with regard to the latter two criteria. It ranked near the middle among subfamilies (5th out of 13) for proportion shared between reared specimens and core Malaise samples (0.093, not significantly different [*P* = 0.89] from the mean among the other 12 subfamilies of 0.095; one-sample *t* test). Likewise, Microgastrinae was typical for completeness, again ranking 5th out of 13, averaged between reared and core Malaise (0.66, not significantly different [*P* = 0.26] from the mean among the other 12 subfamilies of 0.62; one-sample *t* test).

### Step 3. Estimating the Undersampling Ratio for Core Microgastrinae.

In Step 3, we compared the observed core Malaise Microgastrinae to the *Nhat*-1 estimate of true ACG microgastrine richness, based on the pooled Microgastrinae samples (from Step 2). We took this step to estimate the degree to which the observed core Malaise species count is a downward-biased measure of true ACG Microgastrinae richness.

In this step, we assumed that the difference between these measures comprises at least four components: 1) species characteristic of the additional habitats sampled by peripheral Malaise traps (Fig. 1), 2) parasitoid Microgastrinae reared from caterpillars but not captured by a core Malaise trap (Fig. 2), 3) additional (mostly rare) species revealed by barcoded specimens in the much larger sample size of pooled Microgastrinae (21,669 individuals) compared to core Malaise Microgastrinae (only 3,781 individuals), and 4) the estimated 980 species of microgastrines present but not observed in the source assemblages sampled by the estimation procedure that we applied to Microgastrinae (*Nhat*-1 estimate of 2,394 species minus 1,414 observed species).

We wrestled with the “part-whole” taboo of comparing the core Malaise Microgastrinae to pooled Microgastrinae, of which the former is a part. But we concluded that the *Nhat*-1 estimation procedure that we applied to pooled Microgastrinae, before the comparison, was sufficient to exorcise the taboo. And in any case, the objective of the comparison was estimation, not statistical comparison.

### Step 4. Estimating True Species Richness of ACG Insects.

In this step, we adjusted the observed count of core Malaise Insecta upward, to reach an estimate of the true total richness of ACG insects. The first key assumption for this crucial step is that the level of undersampling estimated for Microgastrinae in Step 2 applies approximately to core Malaise insect species richness (as a whole) and that it takes into account the four components of undersampling of ACG species richness enumerated for Step 3, above—but takes them into account for all insects, as exemplified by Microgastrinae.

A second key assumption of this step is that the core Malaise trap data for Insecta are a representative sample of the insect fauna of ACG. Microgastrinae are believed to be well-sampled using Malaise traps, as are most other ichneumonoid parasitoid wasps. In fact, the current standards for Malaise traps are all modifications of the original trap designed by ichneumonologist Henry Townes (60). Students of Braconidae and Ichneumonidae rely heavily, or even exclusively, on this collection method. In contrast, researchers studying other groups of Hymenoptera (e.g., Chalcidoidea, Platygastroidea) often collect using pan-traps and screen-sweeping with nets. Aculeate Hymenoptera (ants, bees, and stinging wasps) are also present, but underrepresented in Malaise traps, and taxonomists of this group tend to collect with sweep nets for bees and wasps, and Winkler extractors and canopy fogging for ants (6, 61). Many species of Aculeata are too visually astute to be fooled into falling into the collection heads of Malaise traps (62).

The bias is more acute when microgastrines are compared with members of other insect orders. The fact that taxonomists studying Coleoptera, Hemiptera, Orthoptera, and other orders do not use Malaise traps as their principal method of collection indicates that these orders are likely to be severely underrepresented in these traps. In effect, all major insect orders other than Diptera and Hymenoptera are likely to be underrepresented in Malaise traps (63). Adjusting for this bias could only increase our estimates of ACG insect richness, and thus our estimate of global insect richness.

To gauge the likely impact on total ACG species richness of limiting our mass sampling to Malaise traps, we compared the proportional composition of the core Malaise captures in our study, by insect orders, to the results of Souto‐Vilarós et al. (64) for the STRI (Smithsonian Tropical Research Institute) rainforest on Barro Colorado Island (BCI) in Panama (just 650 km from ACG) (*SI Appendix*, *Appendix S1*). In a structured, quantitative inventory of major insect orders, Souto‐Vilarós et al. (64) used not only Malaise traps but also six other standard mass sampling techniques (Berlese-Tullgren funnels, Winkler extractors, pitfall traps, beating, polytraps, and light traps), 50 samples each, with all 350 samples fully processed, specimen by specimen, with COI metabarcoding.

Based on the BCI data, Malaise traps were surprisingly effective in capturing most insect orders (*SI Appendix*, Fig. S4). However, using the complete BCI dataset (available online) as a guide, we devised a way to roughly estimate how many additional insect species (BINs) we might have captured in ACG, had we used the full panoply of collecting techniques used in the Panama study, in the same ratios of trap types and trapping effort used in the BCI study (*SI Appendix*, *Appendix S1*).

In brief, insect order by order, we computed a “BCI non-Malaise rate” as the ratio between the total number of species exclusive to BCI non-Malaise sampling methods (numerator) and the number of species captured in BCI Malaise samples (denominator). We then augmented the ACG Malaise total, for each order, by the corresponding BCI non-Malaise rate, scaled by the actual number of species in the ACG core Malaise captures for that order. Summing the augmented ACG values across orders, the emerging total was 144,898 species, 2.7 times the actual number of insect species captured in our core Malaise samples (53,946) (*SI Appendix*, Table S3).

However, as we show in *SI Appendix*, *Appendix S1*, although this result is qualitatively indicative of the limitations of Malaise sampling for several insect orders (*SI Appendix*, Table S3), the numerical values of the ACG order richness, adjusted by the BCI data, are strongly dependent on the design of the BCI study, in which one Malaise trap per subplot was run for 30 d during the wet season and 7 d during the dry season (*SI Appendix*, Fig. S5). Suppose, instead, the Malaise sampling in the BCI study were doubled, but the other six sampling methods remained the same. We built a species accumulation simulation model (*SI Appendix*, *Appendix S1*) to test the effect of adding two of the 50 BCI Malaise samples for every single sample of the other six collection methods. Doubling the Malaise effort in the BCI study, while keeping the other sampling methods unchanged, reduced the adjusted ACG total by 31%, from 144,898 to 99,435 estimated species in simulated core Malaise samples (*SI Appendix*, Fig. S6 and Tables S3 and S4).

Without doubt, including additional collecting methods, beyond Malaise and rearing, would have increased the observed number of insect species in ACG, and thus the estimate of global insect richness. But by how much the estimate would be increased is impossible to guess, with any confidence, based on the Souto‐Vilarós et al. (64) data, given the sensitivity of the result to the sampling design of the BCI study, as conclusively shown by our simulations. The same limitation would surely apply to any other study that combined Malaise with other insect collection methods (e.g., refs. 18 and 65).

Then there is the forest canopy to consider. The BCI study made no attempt to sample the canopy directly. Even within our focal subfamily in the ACG, Microgastrinae, our “pooled” sample (reared plus core Malaise plus peripheral Malaise) is an undercount, most obviously because ACG Malaise traps and rearing both ignore canopy fauna. The insect faunas of cloud forests and rain forests are rich in species (8) that almost never go near the ground unless attracted to light traps. Although it is possible to set Malaise traps on rope systems, in the canopy (63), ACG Malaise traps were placed exclusively at ground level.

Likewise, parataxonomists collecting caterpillars do not sample tree canopies in ACG. It is difficult to estimate the percentage of microgastrine BINs that were missed for this reason, but it could be substantial. Few studies have compared species richness between ground level and canopy—or in intermediate strata—in tropical forests. In research conducted on a tower in Amazonian rainforest, de Souza Amorim et al. (66) concluded that ground-level Malaise traps miss 38.4% of the species of Diptera. Similarly, Basset (67) found that 51% of Diptera species were not found at ground level (0 to 3 m), and Longino and Colwell (61) reported that one-third of the 539 recorded ant species in the La Selva rainforest (Costa Rica) were exclusively found in canopy samples.

We are confident that the collections made in ACG are likely missing a similar proportion of species. Tropical rainforests are not found throughout the world, but they do contain the highest species richness of trees (40, 68) and certainly of insects as well. Although other forest types display different degrees of stratification, correcting this lacuna in our sampling would surely increase our estimates substantially.

### Step 5: Estimating the Global Number of Insect Species.

In our exhaustive search for appropriate groups for upscaling ACG insect richness to global richness, we considered eight candidates: one plant group (trees), three vertebrate groups (amphibians, birds, and mammals), and four insect groups (butterflies, odonates, sphingid moths, and saturniid moths). As the focus of intensive research, worldwide, current global species totals for the birds and mammals are readily available. The ACG totals for birds and mammals are based on informal checklists. The totals for ACG amphibians are based on Edwards et al. (69) and on coauthor Puschendorf’s back-projections for 1940, representing the ACG fauna prior to the amphibian declines of the 1980s–1990s. The 1940 estimate is based on Puschendorf’s field surveys (69, 70), historical museum collections from the Wake and Cannatella 1987 expedition (70), and published inventories. Full rationale and supporting data appear in *SI Appendix*, *Appendix S2*.

The four insect groups we considered for upscaling ratios share the attributes of being large-bodied, colorful, highly mobile, and popular with amateurs as well as professional entomologists—making them strikingly atypical of the vast majority of insects. Considered together, these groups comprise, worldwide, only about 32,000 species, fewer than the 54,000 insect species just from our core ACG Malaise traps. All but the saturniids share another attribute: broad geographical ranges (Dataset S1), making them unsuitable as upscaling reference groups for ACG insects.

The Saturniidae richness estimates are derived from the results of long-term sampling campaigns in ACG, coupling caterpillar rearing, light traps, and DNA barcoding (30, 71), as well as results of collaborative efforts to build a comprehensive DNA barcode reference library for the world fauna of saturniid moths. Thus, in contrast with most other cosmopolitan insect families, they have been studied at ACG with the same tools as the insects captured by core Malaise traps, making them especially suitable for upscaling. Over the past two decades, the integration of DNA barcoding and traditional comparative morphology (complemented by biogeographical, ecological, and behavioral information) has supported the description and systematic revision of these moths by an international community of expert taxonomists, leading to the description of more than 1,500 new taxa (72) and an updated, increasingly accurate estimate of global diversity of saturniid moths.

Why use trees to estimate the richness of insects? Like insects, trees encompass a vast scope of functional roles, demography, dispersal modes, and evolutionary diversification over much of terrestrial Earth. On a local scale—in Panama, like the Souto‐Vilarós study (64)—Basset et al. (12) carried out a comprehensive inventory of tropical rain forest insects (including the forest canopy), conducted by a wide spectrum of traditional methods in a structured protocol, with morphological species identifications or delineations by a large team of experts. A principal finding of that study was a striking correlation between cumulative insect richness and cumulative number of tree species over the 12 study plots.

However, the critical assumption of this step is that the ratio of insect species to tree species—on a global scale—is approximately the same as that ratio for ACG. As pointed out decades ago by Hodkinson and Casson (73), the longstanding concern with host plant specificity for insects (9, 11, 74) that held such leverage on Erwin’s (8) estimates has no direct bearing on our procedure. While host plant specificity is certainly important on local-to-regional scales, broad-scale species richness ratios are all that matter for our estimates.

Global patterns of tree species richness implicitly set the scale for alpha [figure 3 in Cazzolla Gatti et al. (40)] and beta [figure 4 in Cazzolla Gatti et al. (40)] diversity (species turnover) for tree species on a broad geographical scope. Unfortunately, we do not have even such coarse maps for insects, although there has been some progress on a regional scale for tropical herbivorous insects (75, 76).

Despite these considerations, species diversity patterns on a macroscale may allow some qualitative inferences, at least for alpha diversity. The latitudinal gradient in tree species richness is remarkably steep—temperate and high-latitude forest biomes have far fewer tree species, for a given area, than tropical rainforests (68). In contrast, ichneumonoid parasitic wasps have long been suspected to have an atypical latitudinal diversity gradient, with more species (77)—or at least no fewer species (78)—at higher latitudes than in the tropics. The longstanding controversy over this pattern has recently been reviewed by Castellanos‐Labarcena et al. (79). Based on DNA barcode data adjusted by rarefaction and coverage-matching (80) to account for sampling bias, their analysis strongly supports an atypical latitudinal gradient in species richness, with a peak richness at 30 to 60°N. Crucially, this pattern includes not only Ichneumonidae—the subject of Janzen’s provocative study (77)—but also Braconidae, the family of our foundational taxon, the subfamily Microgastrinae. If the ACG ratio of microgastrine wasps to other insects is consistent at higher latitudes, our current global insect estimates—based on tree diversity—are likely too low, assuming even remotely similar patterns of latitudinal species turnover for insects and trees. To the point, a recent global DNA barcode analysis of the 10 most diverse and abundant insect families (three families of Hymenoptera—including Braconidae,_^—^_six families of Diptera, and one of Hemiptera) revealed weak, flat, or inverse latitudinal richness gradients (81).

In further support of this prediction, Hebert et al. (18) barcoded more than one million Canadian insects of 27 orders, yielding nearly 47,000 species (BINs) representing all of Canada—with an asymptotic estimate of 94,000 species, based on lognormal assumptions. With 234 species of trees in Canada (82), the ratio yields 402 insect species per tree species in Canada (94,000/234 = 402), far exceeding the 278 insect species per tree species in our upper estimate for ACG (332,846/1,200 = 277) and the Earth. Similarly, Baker et al. (83) barcoded 892,000 specimens, yielding 42,300 insect species (BINs) representing all of California, with an asymptotic estimate of 61,000 species. With 225 species of trees in California (41), the ratio yields 271 insect species per tree species, comparable to our upper estimate for ACG.

We conclude that latitudinal variation in the ratio of insect species to tree species may bias our global estimate of insect richness, if anything, downward. And, of course, not all terrestrial biomes are forests. Grassland and freshwater ecosystems—only barely present in ACG—have their own insects, which if added to our estimates, would further amplify the global estimate (84).

## Conclusion

Our preferred upscaling reference group, trees, yielded an estimate of global insect richness of 20.3 million, based on the Cazzolla Gatti et al. (40) estimate of global tree richness, adjusted for the likely possibility of spurious one-off identifications. Taken together, the assumptions discussed above render that number—more than three times the current consensus of six million insect species—as far more likely to be an underestimate than an overestimate. Our lower-bound estimates of the global number of insect species based on alternative upscaling reference groups (Fig. 3) averaged 17.3 million—still nearly three times the current consensus.

The magnitude of these estimates rests on the foundation of an underappreciated wealth of parasitoid wasps in the ACG and the tireless work of the entomologists and parataxonomists who study them. The insect data—particularly the long-term Malaise trap data—are one of the few tools we have that can realistically help us track population trajectories of tropical insects in the context of ongoing climate change, habitat modification, and large-scale use of pesticides (85).

## Materials and Methods

### Specimens.

Our data come from three sources. The first source (“core” Malaise samples) comprised insect specimens collected in 15 Malaise traps operated in northwestern Costa Rica’s ACG (http://www.acguanacaste.ac.cr; http://www.gdfcf.org) for a total of 69 Malaise trap-years (Fig. 1, red dots). Each insect specimen from these traps was DNA-barcoded (3), matched to an existing BIN if it existed on BOLD or assigned a new one if it did not fall into an existing BIN, and identified to order, family, and subfamily. We treat BINs as a proxy for species, to obtain species richness estimates.

The 15 core Malaise traps were operated in four localities. Two traps separated by 20 m—one in the open and one in shadow at the forest edge—were set for 2 y in each of the following three ecosystems: Pacific lowland dry forest (Parque Nacional Santa Rosa, Bosque San Emilio, 300 m a.s.l., 10.84389°N, 85.61384°W); montane cloud forest (Estación Cacao, Sendero Derrumbe, 1,300 m a.s.l., 10.93002°N, 85.4617°W); and Caribbean lowland rain forest (Estación San Gerardo, 575 m a.s.l., 10.88009°N, 85.38887°W). The other nine core Malaise traps, each sampled continuously for 7 y, were set across an area of three hectares in an intergrade between rainforest and Pacific dry forest (28). These nine traps were all within a few hundred meters of one another, roughly centered around a trap with the following locality data: 752 m., 10.76248°N, 85.33689°W; the exact georeferenced localities are available on the Barcode of Life Data System (BOLD) (www.boldsystems.org) under the codes PL12-1 to PL12-9. The map in Fig. 1 shows the localities of these core sampling sites, each represented by a red dot. Specimens were harvested from each Malaise trap on a weekly basis.

The core Malaise data include not only parasitoid wasps and other flying hymenopteran species but also the entire scope of insects intercepted by the traps, thus providing a quantitative assemblage context for our richness estimation strategy. All individual insects from the core Malaise traps—regardless of taxon—were barcoded, with about 90% yielding DNA barcode sequences, whereas Hymenoptera were slightly more challenging, with about 85% success (18). Given that the specimens that could not be barcoded may have been additional, otherwise undetected species, these shortfalls in barcoding are another conservative factor for species richness counts. Furthermore, since barcodes for Microgastrine (Hymenoptera) are recovered at a 5% lower rate than nonhymenopterous insects, their richness is relatively underestimated.

The second source of specimens (“peripheral” Malaise samples) comprised an additional 15 Malaise traps, operated since January 2007, throughout ACG. Some of these were operated for several weeks and others for more than a year. The map in Fig. 1 shows the localities of these sampling sites with yellow dots. Not every specimen from these traps was barcoded. Instead, over the years, various taxonomists extracted specimens of their focal taxa from the samples, some of which were barcoded. As explained in the “Strategy” section, for purposes of this study, the only specimens that we are concerned with from peripheral Malaise samples are microgastrine wasps. All microgastrine specimens from the peripheral traps were barcoded, except for a single, common, and visually identifiable species, for which many, but not all specimens were barcoded. Because the richness estimator we employ to combine core, peripheral, and reared data for Microgastrinae (*Nhat*-1, in the CARE-1 package) relies solely on incidence data, this exception has no effect.

The third source of data relies on reared specimens. Daniel Janzen, Winnie Hallwachs, and a 10-to-30-member team of rural parataxonomists have been rearing caterpillars (Lepidoptera larvae) in ACG for more than 40 y (1984–2025) (30, 86, 87). The number of parataxonomists varied from year to year, peaking at about 30 parataxonomists for the middle decades of the project (2000 to 2015), whereas approximately ten parataxonomists were employed near the beginning and end of the rearing project. In the process, they have produced host records for tens of thousands of individual ichneumonoid wasps (parasitoid wasps of the families Ichneumonidae and Braconidae that emerge from caterpillars). In all, these specimens represent 2,618 species of ichneumonoids from 2,967 species of wild-caught caterpillars (e.g., refs. 53 and 88). The sheer number of reared caterpillars (531,453) is unmatched in any other study known to us.

The combination of these three datasets provides the foundation for our global estimates of species richness. As of December 2024, nearly 2.5 million specimens, nearly all of them insects, have been barcoded from ACG, representing more than 93,000 BINs—less than a third of our statistical estimate of nearly 333,000 insect species for ACG.

### Barcode Analysis.

From 2013 to 2021, 1,930,021 Malaise-trapped specimens from the 15 core traps in the ACG were DNA barcoded at the Centre for Biodiversity Genomics at the University of Guelph, Canada. Every specimen in each of the 3,588 weekly samples (mean = 538 specimens) was analyzed, except a few very abundant morphospecies. For the latter taxa, 5 to 24 specimens were analyzed while the others were set aside. The specimens in each sample were first partitioned into two size categories. Small individuals were placed directly into a well in a 96-well plate, while large-bodied specimens were pinned before a small tissue sample was removed and placed into a well. These two workflows meant that DNA extracts from small specimens were whole body extracts while those from large specimens were typically a leg extract.

Because DNA was extracted nondestructively, all voucher specimens remain available for morphological analysis. The DNA extracts were immediately transferred to –20 °C, where they formed a resource for subsequent barcode recovery. Each specimen was photographed and the image linked to its sequence record on BOLD (89, 90) together with key metadata (e.g., place and time of collection, taxonomic assignment).

Sequence analysis was initiated by PCR amplification of the 658 base-pair barcode region of cytochrome *c* oxidase 1 (COI), using a standard thermocycling regime and primers (91). From 2013 until early 2018, all PCR products were Sanger sequenced on an ABI 3730XL, but subsequent analyses employed Sequel or Sequel II platforms. Specimens processed via Sanger delivered either a single barcode sequence or an uninterpretable trace file, while nearly all specimens processed on Sequel generated multiple reads. When divergences among the sequences from a specimen were small, they were grouped into a single OTU, but when divergences were higher than 2.3%, they were partitioned into two or more. The dominant OTU ordinarily originated from the source specimen, while secondary OTUs often reflected the recovery of endosymbionts, NUMTs, parasites, or parasitoids. These nontarget sequences were retained but were placed in “quarantine” on BOLD. After the barcode sequence for a specimen was uploaded to BOLD, it was assigned a BIN (92). In total, 1,653,327 specimens (85.7% of those analyzed) from the ACG core Malaise traps received a BIN assignment and formed the basis for subsequent analyses.

Specimens from the 15 peripheral Malaise traps were treated in the same way. Of the total of 6,515 specimens from the peripheral traps, 4,593 were Sanger sequenced and 1,922 went through the Sequel pipeline.

### Analysis.

All analysis was done in R (93), with custom code, incorporating function libraries SpadeR (94), CARE-1 (34), dplyr, stingr (95), pairwiseCI (96), and vegan (97). Hierarchical modeling was done with brms (98). Strategy and details of the analysis are specified in the main text, as they are fundamental to understanding our work.

## Supplementary Material

Appendix 01 (PDF)

Dataset S01 (XLSX)

## Data Availability

All data and annotated code are publicly available at https://github.com/lmguzman/world_insect_richness and are permanently archived in Zenodo 10.5281/zenodo.18943734. ACG Saturniidae data can be downloaded from https://ln5.sync.com/dl/e4aea21b0/8z5snjmg-d4ewrbez-uzz3bnbm-7sgrv4cq. GBIF Saturniidae data can be downloaded from https://ln5.sync.com/dl/3cca04c30/pnb2j7ky-ycrvgpbm-dejz8azu-7bcjr363. Data used in this manuscript include previously published data: Insect trapping by Souto‐Vilarós (64): https://doi.org/10.5281/zenodo.16586355. All other data are included in the manuscript and/or supporting information.
