# Supplementary material for: Constructing a lower-bound estimate of the global number of insect species on a hyperdiverse empirical foundation
Source: Proc Natl Acad Sci U S A. 2026 Jun 29;123(27):e2524283123. doi: 10.1073/pnas.2524283123 (PMC13342947; doi:10.1073/pnas.2524283123)
Supplement: Supplementary file 1 — Appendix 01 (PDF) [file pnas.2524283123.sapp.pdf]

PNAS

Supporting Information for:

Constructing a lower-bound estimate of the global number of insect species on a hyperdiverse empirical foundation

Robert K. Colwell[a,b], Laura Melissa Guzman[c], Dirk Steinke[d], Anne Chao[e], Daniel H. Janzen[f], Winnie Hallwachs[f], Austin Baker[g], José L. Fernández-Triana[h], Paul D.N. Hebert[d], Frank Joyce[i], Robert Puschendorf[j]; Donald L. J. Quicke[k], Rodolphe Rougerie[l]; M. Alex Smith[m], Nelson Zamora[n], Michael J. Sharkey[n,o]

To whom correspondence may be addressed. Email: lmg343@cornell.edu

Author affiliations: [a] Department of Ecology and Evolutionary Biology, University of Connecticut, Storrs, CT 06269-3043; [b] Entomology Section, University of Colorado Museum of Natural History, Boulder, CO 80309-0001; [c] Department of Entomology, Cornell University, Ithaca, NY 14853-2601; [d] Centre for Biodiversity Genomics, Department of Integrative Biology, University of Guelph, Guelph, ON N1G2W1, Canada; [e] Institute of Statistics, National Tsing Hua University, Hsin-Chu 30043, Taiwan; [f] Department of Biology, University of Pennsylvania, Philadelphia, PA 19104-6018; [g] Department of Entomology, Natural History Museum of Los Angeles County, Los Angeles, CA 90007; [h] Canadian National Collection of Insects, Ottawa, ON K1A 0C6, Canada; [i] Monteverde Institute, Monteverde, Puntarenas, 60109, Costa Rica; [j] School of Biological and Marine Sciences, University of Plymouth, Plymouth PL4 8AA, United Kingdom; [k] Center of Excellence in Integrative Insect Ecology, Department of Biology, Faculty of Science, Chulalongkorn University, Bangkok 10330, Thailand; [l] Department of Origins and Evolution, Institut de Systématique, Évolution, Biodiversité, Muséum National d'Histoire Naturelle, Centre National de la Recherche Scientifique, École Pratique des Hautes Études – Université Paris Sciences & Lettres, Sorbonne Université, Université des Antilles, Paris F-75005, France; [m] Department of Integrative Biology, University of Guelph, Guelph, ON N1G2W1, Canada; [n] Escuela de Ingeniería Forestal, Instituto Tecnológico de Costa Rica, Cartago 159-7050, Costa Rica; [o] The Hymenoptera Institute, Lexington, KY 40502.

To whom correspondence may be addressed. Laura Melissa Guzman
Email: lmg343@cornell.edu

This PDF file includes:

(Each entry below is internally hyperlinked)

Table S1. Estimated ACG (Área de Conservación Guanacaste) and global richness for Hymenoptera, Ichneumonoidea, and Braconidae, anchored in the 1500-species estimate of ACG tree species richness and the conservative estimate of global tree richness (73,274 species) of (1).

Table S2. Comparing richness, completeness, and shared species for reared specimens vs. core Malaise samples for 13 ichneumonoid subfamilies (Hymenoptera: Ichneumonidae, Braconidae).

Table S3. Computational steps to estimate the number of additional species that might have been captured with non-Malaise methods in the ACG, had we used the full panoply of collecting techniques used in the BCI study, in the same ratios of trap types and trapping effort.

Table S4. Computational steps to estimate the number of additional species that might have been captured with non-Malaise methods in the ACG, had we used the full panoply of collecting techniques used in the BCI study, but doubled the Malaise effort in the BCI data.

Figure S1. Non-metric multidimensional scaling (NMDS) plots for two subfamilies of Hymenoptera and two subfamilies of Diptera collected by rearing of caterpillars or by Malaise traps in the Cacao sector of ACG.

Figure S2. Richness upscaling factor (global species richness divided by ACG species richness) as a function of median geographical range size for eight candidate groups for upscaling (bottom table in Dataset S1, Spreadsheet).

Figure S3. Distance decay of similarity (proportion shared species) between all pairs of all 27 ACG Malaise traps. Blue (lower) regression line fits all points; red (upper) regression line fits all non-zero points

Figure S4. Probability that an insect recorded in the Souto-Vilarós (BCI) study (2) will be captured by a Malaise trap, rather than by any of the other six mass sampling techniques applied in the study.

Figure S5. Simulation results for the BCI study (2) as actually conducted.

Figure S6. Hypothetical simulation results for the BCI study (2), doubling the Malaise effort in the BCI study, while keeping the other sampling methods unchanged.

Figure S7. Amphibian species richness in Área de Conservación Guanacaste (ACG), northwestern Costa Rica, before and after the amphibian declines of the 1980s–1990s.

Figure S8. Amphibian species richness at Estación Cacao (Volcán Cacao, ACG cloud forest, ~1,300 m a.s.l.) before and after the amphibian declines of the 1980s–1990s.

Figure S9. Cumulative amphibian species descriptions for Costa Rica, 1835–2025.

Figure S10. Cumulative salamander (Caudata) species descriptions for Costa Rica.

Appendix S1. Comparing the composition of ACG core Malaise captures, in our study, to the results of Souto-Vilarós (2) for Malaise and six other collection methods on Barro Colorado Island (BCI) to assess the potential effect of using additional collection methods in the ACG.

Appendix S2. Basis for the ACG amphibian species richness estimate for approximately 1940 used in the global upscaling analysis.

Dataset S1. Analysis of candidate groups for upscaling ACG insect richness to global insect richness. (Excel spreadsheet. Separate file.)

SI References

Table S1. Estimated ACG (Área de Conservación Guanacaste) and global richness for Insecta, Hymenoptera, Ichneumonoidea, and Braconidae, anchored in the 1200-species estimate of ACG tree species richness and the conservative estimate of global tree richness (73,274 species) of (1).

Taxon	Core Malaise	ACG (est)	ACG CI Low	ACG CI High	Global (est)	Global CI Low	Global CI High
Insecta	53,945	332,846	306,847	364,963	20,324,144	18,736,586	22,285,246
Hymenoptera	13,934	85,974	79,259	94,270	5,249,729	4,839,662	5,756,282
Ichneumonoidea	3,402	20,991	19,351	23,016	1,281,727	1,181,608	1,405,402
Braconidae	2,160	13,327	12,286	14,613	813,795	750,228	892,319
Microgastrinae	388	2,394	2,207	2,625	146,182	134,763	160,287

Table S2. Comparing richness, completeness, and shared species for reared specimens vs. core Malaise samples for 13 ichneumonoid subfamilies. **Braconidae:** Microgastrinae, Agathidinae, Cheloninae, Euphorinae, Macrocentrinae, Orgilinae, Rogadinae. **Ichneumonidae:** Anomaloninae, Campopleginae, Mesochorinae, Metopiinae, Ophioninae, Tryphoninae.

Subfamily	Total Species	Rank total Species	Species Reared	Rank Species Reared	iChao1 Reared	Completeness Reared	Rank Completeness Reared
Microgastrinae	1168	1	889	1.0	1131	0.79	4.0
Campopleginae	333	2	243	2.0	298	0.82	3.0
Cheloninae	190	3	77	7.0	124	0.62	11.5
Euphorinae	186	4	58	9.0	78	0.74	7
Mesochorinae	184	5	151	3.0	218	0.69	9
Rogadinae	168	6	98	6.0	157	0.62	11.5
Agathidinae	150	7	141	4.0	168	0.84	2
Ophioninae	108	8	101	5.0	131	0.77	5
Metopiinae	88	9	63	8.0	87	0.72	8
Macrocentrinae	57	10	41	11	117	0.35	13
Orgilinae	55	11	28	12	32	0.88	1
Anomaloninae*	50	12	44	10	59	0.75	6
Tryphoninae*	31	13	12	13	18	0.67	10
**Mean**			150		201	0.71	
**SD**			231		290		
**Total**	2768						

Subfamily	Species Core Malaise	Rank Species Core Malaise	iChao1 Core Malaise	Complete-ness Core Malaise	Rank Complete-ness Core Malaise	Mean Completeness	Mean Completeness Rank
Microgastrinae	388	1.0	725	0.54	9.0	0.66	5.0
Campopleginae	161	2.0	270	0.60	4.0	0.71	3.0
Cheloninae	117	4.0	278	0.42	11.0	0.52	11.0
Euphorinae	136	3.0	213	0.64	3.0	0.69	4.0
Mesochorinae	48	6.0	83	0.58	6.0	0.64	8.0
Rogadinae	79	5.0	122	0.65	2.0	0.64	9.0
Agathidinae	32	7.0	46	0.70	1.0	0.77	1.0
Ophioninae	23	11.0	42	0.55	8.0	0.66	6.0
Metopiinae	30	8.5	82	0.37	12.0	0.54	10.0
Macrocentrinae	26	10.0	57	0.46	10.0	0.41	13.0
Orgilinae	30	8.5	51	0.59	5.0	0.74	2.0
Anomaloninae*	10	13.0	18	0.56	7.0	0.66	7.0
Tryphoninae*	21	12.0	63	0.33	13.0	0.50	12.0
**Mean**	85		158	0.54		0.63	
**SD**			192	0.11		0.10	
**Total**	**1186**						

Subfamily	Reared only	Core only	Both	Total Species	Proportion shared	Rank proportion shared
Microgastrinae	780	279	109	1168	0.093	5
Campopleginae	172	90	71	333	0.213	1
Cheloninae	73	113	4	190	0.021	13
Euphorinae	50	128	8	186	0.043	12
Mesochorinae	136	33	15	184	0.082	7
Rogadinae	89	70	9	168	0.054	11
Agathidinae	118	9	23	150	0.153	3
Ophioninae	85	7	16	108	0.148	4
Metopiinae	58	25	5	88	0.057	9
Macrocentrinae	31	16	10	57	0.175	2
Orgilinae	25	27	3	55	0.055	10
Anomaloninae*	40	6	4	50	0.080	7
Tryphoninae*	10	19	2	31	0.065	8
**Mean**					0.095	
**SD**					0.058	
**Total**	**1667**	**822**	**279**	**2768**		

* Only Lepidoptera parasitoids are included for Tryphoninae and Anomaloninae.

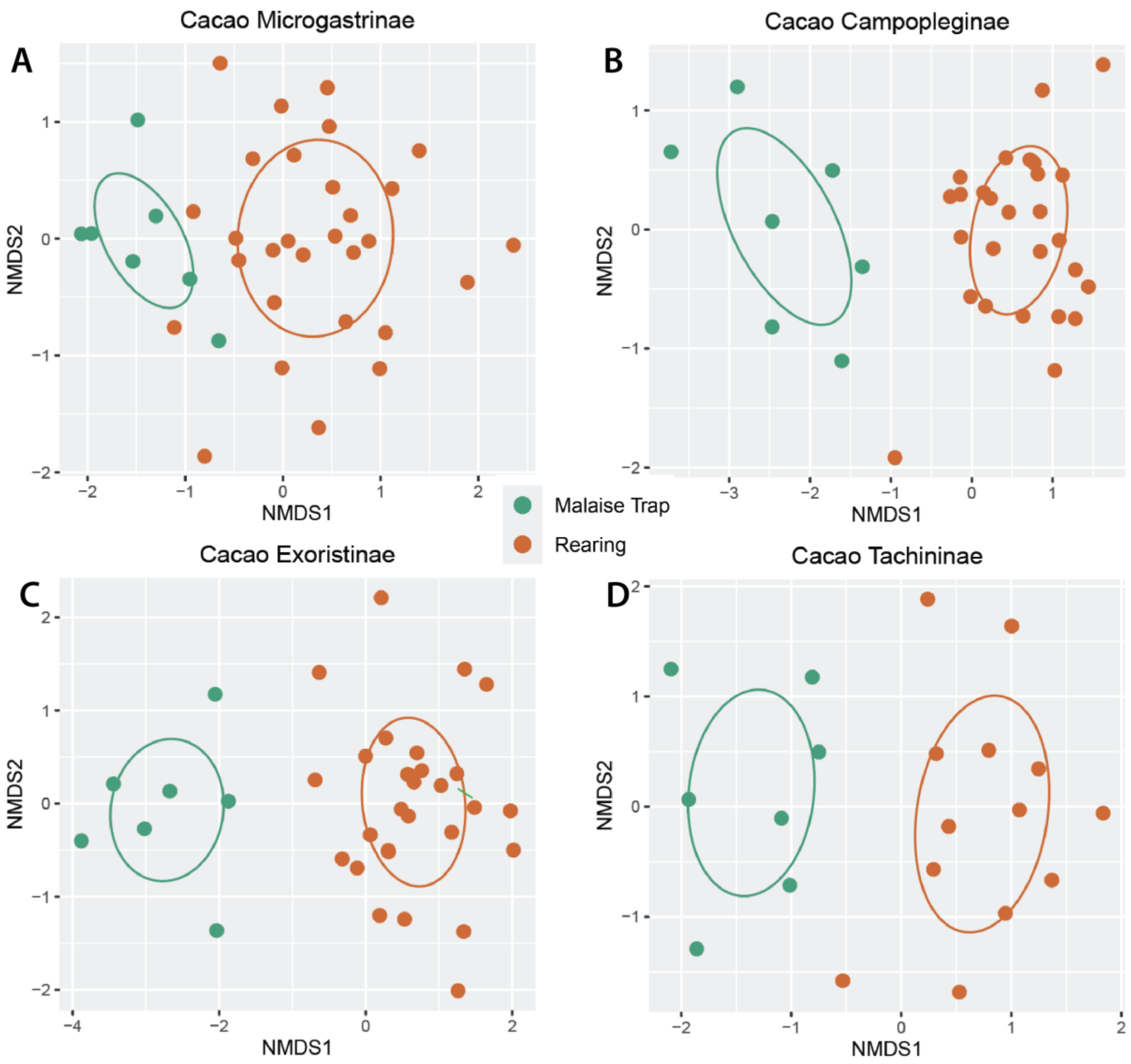

Figure S1. Non-metric multidimensional scaling (NMDS) plots for two subfamilies of Hymenoptera and two subfamilies of Diptera collected by rearing of caterpillars or by Malaise trap in the Cacao sector of ACG. Ordinations are based on the Chao index calculated between sites (3). The Cacao sector covers an ~1000 m elevational gradient from the Gongora rearing station in a mix of dry and rain forest at ~500 m, past the Cacao station at 1000 m, and into cloud forest at the top of the volcano. In each case, the communities of parasitic wasps and flies in these forests (collected indirectly by parataxonomists when they collected host species of lepidopteran caterpillars, or directly by Malaise trap—a subset of the traps in Figure 1 in the Main Text), are significantly different (Permanova). **(A)** Microgastrinae (2,443 individuals from 37 sites and 324 species. *Stress* = 0.119. F = 2.293, *df* = 1, *p* < 0.001). **(B)** Campopleginae (1,052 individuals from 36 sites and 110 species. *Stress* = 0.98. *F* = 6.97, *df* = 1, *p* < 0.001). **(C)** Exoristinae (1,628 individuals from 26 sites and 266 species. *Stress* = 0.109. *F* = 5.004, *df* = 1, *p* < 0.001). **(D)** (Tachininae 420 individuals from 20 sites and 84 species. *Stress* = 0.061. *F* = 3.45, d*f* = 1, *p* < 0.001) (4).

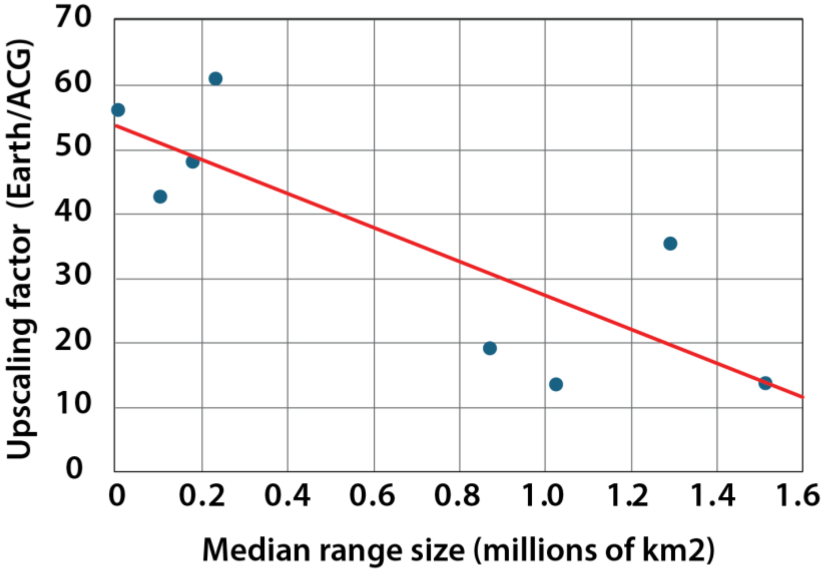

Figure S2. Richness upscaling factor (global species richness divided by ACG species richness) as a function of median geographical range size for eight candidate groups for upscaling (bottom table in Dataset S1, Excel spreadsheet); $R^2 = 0.6742$, $|r| =$ effect size $= 0.821$.

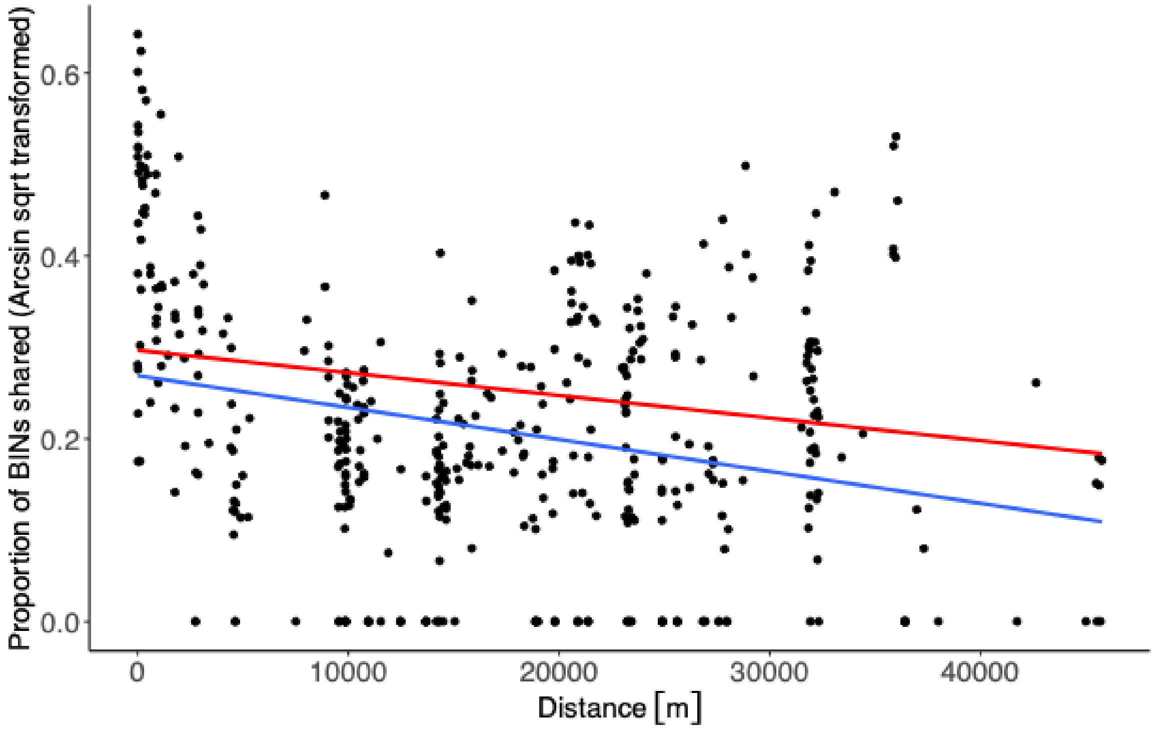

Figure S3. Distance decay of similarity (proportion shared microgastrine species) between all pairs of all 27 ACG Malaise traps. Blue (lower) regression line fits all points; red (upper) regression line fits all non-zero points.

Appendix S1. Comparing the composition of ACG core Malaise captures, in our study, to the results of Souto-Vilarós (2) for Malaise and six other collection methods on Barro Colorado Island (BCI) to assess the potential effect of using additional collection methods in the ACG.

The R code for all computations and figures is available at https://github.com/lmguzman/world_insect_richness.

As briefly outlined in the Main Text, to gauge the likely impact on total ACG species richness of limiting our mass sampling to Malaise traps, we compared the proportional composition of the core Malaise captures in our study, by insect orders, to the results of (2) for the STRI (Smithsonian Tropical Research Institute) rainforest on Barro Colorado Island (BCI) in Panama. In a structured, quantitative inventory of major insect orders, Souto-Vilarós and colleagues (2) used not only Malaise traps, but also six other standard mass sampling techniques (Berlese-Tullgren funnels, Winkler extractors, pitfall traps, beating, polytraps, and light traps), 50 samples each, with all 350 samples fully processed, specimen by specimen, with COI metabarcoding.

In the BCI study (based on the complete BCI dataset, available online), Malaise traps, alone, were surprisingly effective in capturing nearly all insect orders within a narrow range of probabilities, in the context of all seven mass sampling techniques (Figure S4).

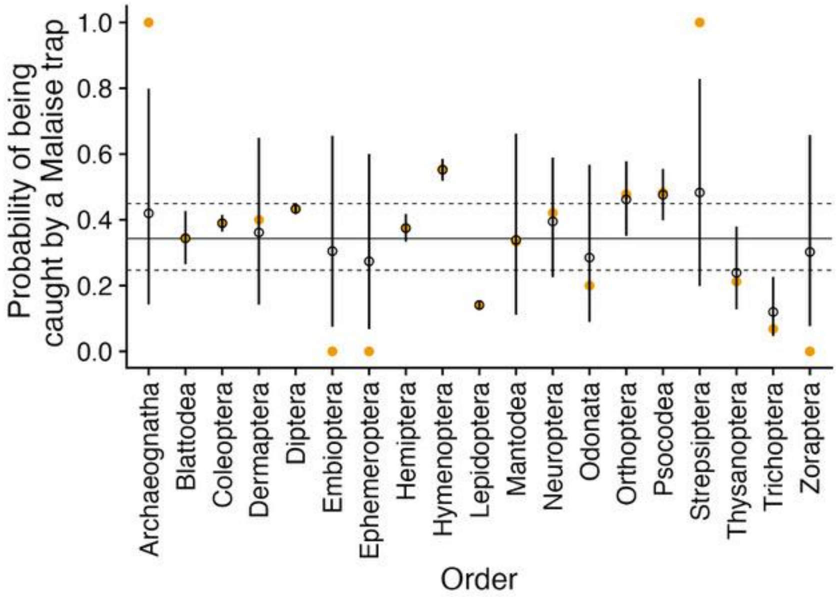

Figure S4. Probability that an insect recorded in the Souto-Vilarós study (2) will be captured by a Malaise trap, rather than by any of the other six mass sampling techniques applied in the study. Orange filled circles are based on the raw data. Open circles emerge from a Bayesian generalized multivariate, multilevel, binomial model, with logit links and insect orders as levels. The dashed lines show the 95% credible interval for the multi-level mean. Among the common orders, Hymenoptera are substantially more likely, and Lepidoptera less likely to be captured by Malaise than by other collection methods.

Using the complete BCI dataset as a guide, we devised a way to roughly estimate how many additional insect species (BINs) we might have captured in ACG, had we used the full panoply of collecting techniques used in the BCI study, in the same ratios of trap types and trapping effort used in the BCI study. Table S3 shows the steps (as columns) taken to estimate the number of additional species that might have been captured with non-Malaise methods in the ACG and adjusted totals. A narrative description follows the table.

Table S3. Computational steps to estimate the number of additional species that might have been captured with non-Malaise methods in the ACG, had we used the full panoply of collecting techniques used in the BCI study, in the same ratios of trap types and trapping effort.

Order	*Total:* BCI number BINs per order	BCI number BINs Malaise	BCI number BINs not Malaise = (Unique to other methods)	(BCI Unique to other methods) per (BCI Malaise species)	ACG core Malaise	Additional species in ACG total if all non-Malaise BCI methods were used in ACG	ACG Malaise plus estimated non-Malaise species
**Archaeognatha**	1	1	0	0.000	18	0	18
**Blattodea**	119	41	78	1.902	186	354	540
**Coleoptera**	1413	551	862	1.564	6844	10707	17551
**Dermaptera**	5	2	3	1.500	31	47	78
**Diptera**	3611	1565	2046	1.307	24528	32067	56595
**Embioptera**	1	0	1	0.000	4	0	4
**Ephemeroptera**	2	0	2	0.000	7	0	7
**Hemiptera**	514	193	321	1.663	2679	4456	7135
**Hymenoptera**	834	462	372	0.805	13934	11220	25154
**Lepidoptera**	2733	383	2350	6.136	4455	27335	31790
**Mantodea**	3	1	2	2.000	23	46	69
**Mecoptera**	0	0	0	0.000	1	0	1
**Neuroptera**	19	8	11	1.375	86	118	204
**Odonata**	5	1	4	4.000	1	4	5
**Orthoptera**	67	32	35	1.094	320	350	670
**Phasmatodea**	0	0	0	0.000	18	0	18
**Plecoptera**	0	0	0	0.000	8	0	8
**Psocodea**	149	72	77	1.069	297	318	615
**Strepsiptera**	2	2	0	0.000	3	0	3
**Thysanoptera**	33	7	26	3.714	286	1062	1348
**Trichoptera**	44	3	41	13.667	210	2870	3080
**Zoraptera**	1	0	1	0.000	2	0	2
**Zygentoma**	0	0	0	0.000	5	0	5
**Totals**	**9,556**		**6,232**		**53,946**	**90,952**	**144,898**

Insect order by order, we computed a "BCI non-Malaise rate" as the ratio between the total number of BCI species exclusive to non-Malaise sampling methods (numerator) and the number of species captured in BCI Malaise samples (denominator). We then augmented the ACG Malaise total, for each order, by the corresponding BCI non-Malaise rate, scaled by the actual number of species in the ACG core Malaise captures for that order. Summing the augmented ACG values across orders in Table S2, the emerging total was 144,866 species, 2.7 times the actual number of insect species captured in our core Malaise samples (53,945).

Although this result is qualitatively indicative of the limitations of Malaise sampling for several insect orders, the numerical values of ACG order richness, adjusted by the BCI data, are strongly dependent on the design of the BCI study, in which one Malaise trap per subplot was run for 30 days during the wet season and 7 days during the dry season. We show this effect by simulation.

A simulation model to assess the stability of the ratio (BCI-Unique-to-Other)/(BCI Malaise) as samples accumulate

Conceptual algorithm:

(1) Choose one of the 50 Malaise samples, at random without replacement. Keep its list of *N* species names (or codes) captured in the first column of an *N*-by-50 matrix called "Accumulated Malaise."

(2) Choose one of the 50 non-Malaise samples, for each of the 7 non-Malaise collection methods, at random without replacement. Pool the 7 samples of species captured and place them in the first column of an *N*-by-50 matrix called "Acccumulated Non-Malaise."

(3) Compare the Accumulated Malaise with Accumulated Non-Malaise species lists and delete any matches from the Non-Malaise list, to create the first column of an *N*-by-50 matrix called "Accumulated Unique to Non-Malaise."

(4) Repeat steps 1 to 3 (49 times) until all 50 samples for all methods have been accumulated, placing the successive results in successive columns of the N by 50 matrices. In other words, *e.g.*, Column *j* is the list from Column (*j* – 1) with species new to step *j* added.

(5) Plot the species accumulation curves for Malaise and Unique-to-Non-Malaise (the incidence totals for their successive 50 columns).

(6) Re-run the entire simulation 100 times, and plot the mean curves, to get smooth curves.

Results of the simulations

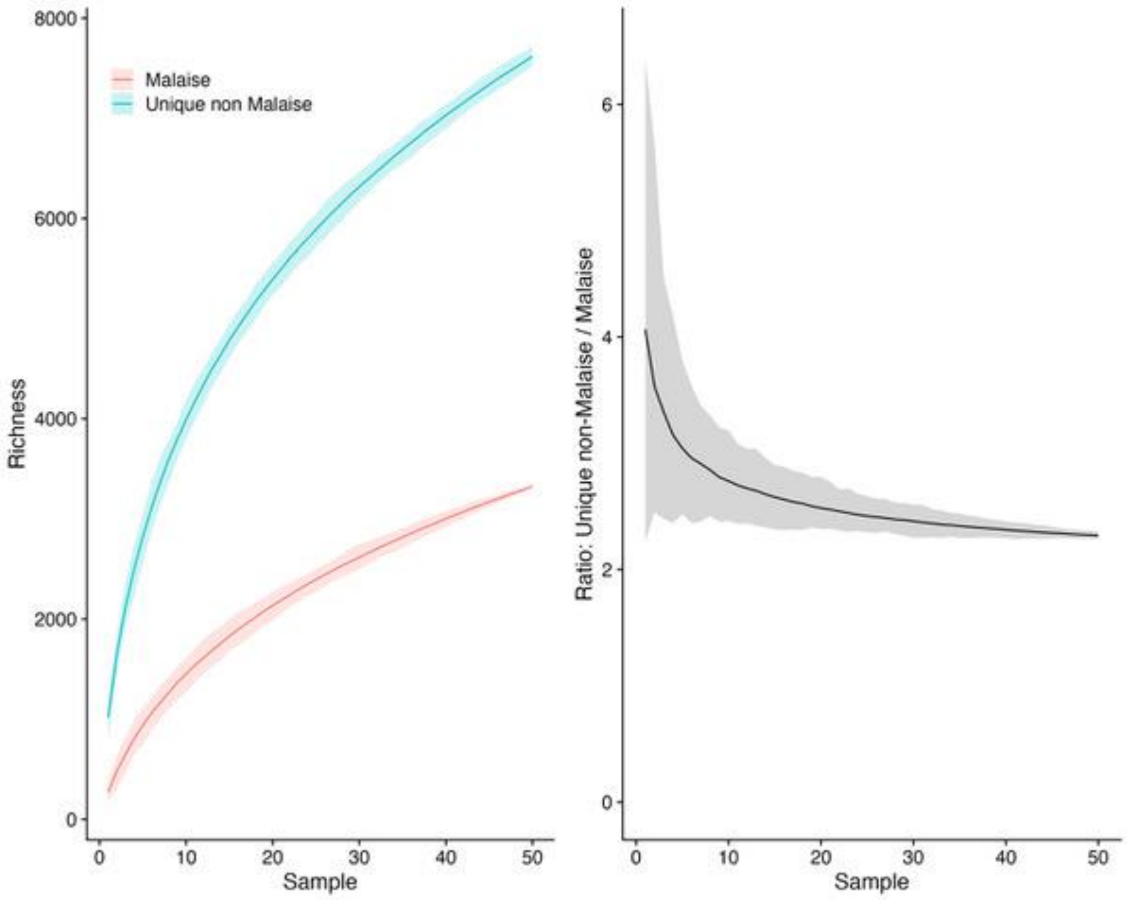

Figure S5. Simulation results for the BCI study (2) as actually conducted. Sample accumulation is randomized, with a single randomly chosen Malaise sample in each step, contrasted with one set of randomly chosen samples (pooled) from each of the other six collecting methods, counting only species that were missed by the Malaise samples. Sampling is without replacement. The panels show the mean results for 100 iterations of the procedure. Envelopes show 0.025 and 0.975 quantiles among iterations.

In Figure S5, the ratio between unique-to-non-Malaise (numerator) and Malaise (denominator) levels off at approximately 2.32. For the procedure as actually run in the BCI (2) study, Malaise traps, alone, captured fewer than half as many insect species as the number of species captured by the other six collecting methods, pooled, that were missed by the Malaise samples.

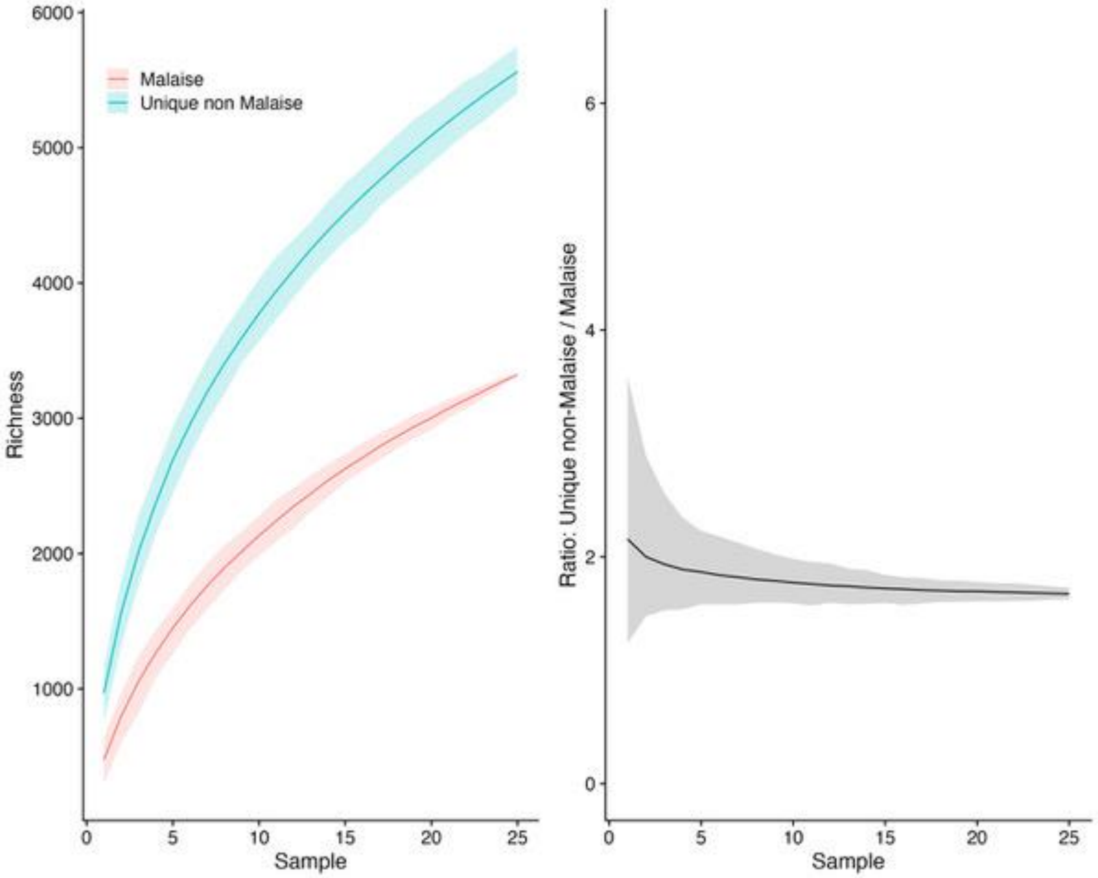

Figure S6. Hypothetical simulation results for the BCI study (2), doubling the Malaise effort in the BCI study, while keeping the other sampling methods unchanged. Sample accumulation is randomized, with two randomly chosen Malaise samples (from the study) pooled in each step, contrasted with one set of randomly chosen samples (pooled) from each of the other six collecting methods, counting only species that were missed by the Malaise samples. The panels show the mean results for 100 iterations of the procedure. Envelopes show 0.025 and 0.975 quantiles among iterations. Because sampling is without replacement, and the study produced only 50 Malaise samples, the doubling results in the simulation ending at 25 accumulated (double) samples—relying solely on the actual BCI data.

In Figure S6, the ratio between unique-to-non-Malaise (numerator) and Malaise (denominator) levels off at approximately 1.71, for this hypothetical doubled Malaise sampling design, compared with 2.32 in Figure S5, for the sampling as actually conducted in the BCI study (2).

In Table S4, below, we estimate the effect of doubling the Malaise effort in the BCI study on adjusted ACG core Malaise capture, while keeping the sampling rate of the six non-Malaise BCI sampling methods constant, following the same logic as in Table S3.

Table S4. Computational steps to estimate the number of additional species that might have been captured with non-Malaise methods in the ACG, had we used the full panoply of collecting techniques used in the BCI study, but doubled the Malaise effort in the BCI data. See Figure S4. For every random pair of (empirical) Malaise samples pooled, only one random set of non-Malaise samples is accumulated, so only half of the BCI non-Malaise sample sets are used.

Order	***Total:*** **BCI number BINs per Order**	**BCI number BINs Malaise**	**Number of BCI BINs *not* Malaise = (Unique to other methods)**	**Half of number of BCI BINs not Malaise = (Unique to other methods)**	**(Half of BCI unique to other methods) per (BCI Malaise species)**	**ACG core Malaise**	**Additional species in ACG total if all non-Malaise BCI methods were used in ACG**	**ACG Malaise plus estimated non-Malaise species**
**Archaeognatha**	1	1	0	0	0.000	18	0	18
**Blattodea**	119	41	78	39	0.951	186	177	363
**Coleoptera**	1413	551	862	431	0.782	6844	5353	12197
**Dermaptera**	5	2	3	1.5	0.750	31	23	54
**Diptera**	3611	1565	2046	1023	0.654	24528	16041	40569
**Embioptera**	1	0	1	0.5	0.000	4	0	4
**Ephemeroptera**	2	0	2	1	0.000	7	0	7
**Hemiptera**	514	193	321	160.5	0.832	2679	2229	4908
**Hymenoptera**	834	462	372	186	0.403	13934	5615	19549
**Lepidoptera**	2733	383	2350	1175	3.068	4455	13668	18123
**Mantodea**	3	1	2	1	1.000	23	23	46
**Mecoptera**	0	0	0	0	0.000	1	0	1
**Neuroptera**	19	8	11	5.5	0.688	86	59	145
**Odonata**	5	1	4	2	2.000	1	2	3
**Orthoptera**	67	32	35	17.5	0.547	320	175	495
**Phasmatodea**	0	0	0	0	0.000	18	0	18
**Plecoptera**	0	0	0	0	0.000	8	0	8
**Psocodea**	149	72	77	38.5	0.535	297	159	456
**Strepsiptera**	2	2	0	0	0.000	3	0	3
**Thysanoptera**	33	7	26	13	1.857	286	531	817
**Trichoptera**	44	3	41	20.5	6.833	210	1435	1645
**Zoraptera**	1	0	1	0.5	0.000	2	0	2
**Zygentoma**	0	0	0	0	0.000	5	0	5
**Totals**	**9,556**		**6,232**	**3,116**		**53,946**	**45,489**	**99,435**

Doubling the Malaise effort in the BCI study, while keeping the other sampling methods unchanged, reduced the adjusted ACG total from 144,898 species (Table S3) to 99,435 species (Table S4)—a 31% reduction. This effect, approximate though it is, undermines any confidence in the quantitative results of Table S2.

Without doubt, however, including additional collecting methods (beyond Malaise and rearing) would have increased the observed total number of insect species in ACG, and thus the estimate of global insect richness. But by how much is impossible to estimate with any confidence, based on the BCI (2) data, given the sensitivity of the result to the sampling design of the BCI study, as conclusively shown by our simulations.

Appendix S2. Basis for the ACG amphibian species richness estimate for approximately 1940 used in the global upscaling analysis

Rationale

This appendix documents the basis for the pre-decline ACG amphibian species richness estimate of approximately 160 species used for ~1940 in the global insect upscaling analysis. The term "~1940" refers to a biological baseline, not a census conducted in that year. It is intended to represent the amphibian fauna of the Área de Conservación Guanacaste (ACG) before the major amphibian declines of the 1980s–1990s substantially reduced the modern assemblage, and before routine molecular taxonomy began to resolve cryptic diversity in this group. The reconstruction is grounded in three sources: co-author Robert Puschendorf's post-decline field surveys (1999–2023)(5, 6)), historical museum collections from the 1987 UC Berkeley expedition led by D. B. Wake and D. Cannatella (6), and published inventories (5). An empirically verified minimum of 69 ACG amphibian species is documented here. The purpose of this appendix is to demonstrate that this verified minimum is a conservative floor, and that the pre-decline estimate of ~160 species used in the upscaling is biologically justified.

One aspect of the upscaling methodology deserves brief explanation here. ACG amphibian richness appears in the denominator of the upscaling equation: a larger pre-decline estimate therefore yields a more conservative, smaller, global insect richness projection. The estimate of ~160 species used in Appendix S2, applying a 2× correction to the published museum-record baseline of 80 historical ACG species (5) is accordingly the conservative choice for a paper committed to lower-bound estimation of global insect richness, throughout. For comparison, restricting the amphibian denominator to the current post-decline observed count would produce a global insect estimate roughly twice as large, decidedly inconsistent with projections from other upscaling reference groups.

Amphibian declines in Costa Rica and ACG

Costa Rica is one of the best-documented Neotropical countries for amphibian declines. These population collapses, concentrated largely in montane communities, reverberated through Costa Rica from the late 1980s onward (7-11). Several species disappeared, most famously the golden toad *Incilius periglenes* from Monteverde (8). Some species classified as Extinct were subsequently rediscovered, including *Incilius holdridgei* (12), but these surviving populations are small and fragmented remnants of a formerly much richer montane fauna coinciding with some of Costa Rica's principal centers of endemism.

Critically, these declines unfolded largely before molecular methods became routine in amphibian taxonomy, so many populations, and the cryptic diversity they harboured, were lost before sequence-based approaches could resolve them. The figures below place this timing in broader context and document the consequences for the ACG pre-decline estimate.

The empirical basis for the pre-decline estimate

Sixty-nine species is the empirically verified post-decline minimum, reconstructed from R. Puschendorf's field surveys (1999–2023), the Wake and Cannatella museum material, other published surveys (5), with taxonomy following Frost (13). Of these, 59 are currently

detectable, 8 are locally extirpated, and 2 are data deficient. This is a documented minimum, not a census.

Edwards *et al.* (5) state in their Discussion: "Historic museum records kept at the Museo de Zoología, Universidad de Costa Rica have documented 80 species, consisting of 75 Anurans, one Gymnophiona and four Caudata within ACG." This figure appears in the primary ACG amphibian inventory on which the upscaling is based and already substantially exceeds the post-decline reconstruction, without any additional analysis.

Starting from the published museum-record baseline of 80 species, we apply a 2× correction, the same loss factor used throughout this study, to account for three independent sources of underestimation documented in the sections below: the spatial incompleteness of pre-decline surveys across ACG's 126,000 ha, which span Pacific lowland dry forest, coastal and mangrove habitats on the Santa Elena Peninsula, premontane transitional forest, montane cloud forest across three volcanic massifs (Volcán Cacao, Volcán Orosí, and Volcán Rincón de la Vieja), and Caribbean lowland rainforest—a landscape of exceptional ecological and geological heterogeneity in which only the most accessible portions of Volcán Cacao and the Santa Rosa sector received any systematic herpetological attention before the declines; the extensive cryptic diversity in plethodontid salamanders that was invisible to morphological taxonomy before 2000; and the absence of any systematic molecular sequencing from pre-decline survey work.

ACG amphibian diversity before and after the declines

Pre-decline herpetological surveys in ACG were opportunistic expeditions concentrated at a small number of accessible localities, principally Volcán Cacao, Santa Rosa, and a handful of accessible streams. The upper elevations of Volcán Orósi, most of Volcán Cacao and the Volcán Rincón de la Vieja complex, including large portions of the other ecosystems present in ACG were never systematically surveyed before the collapse at the end of the 1980's. Any species present in these unsurveyed areas would be entirely absent from the historical record regardless of methodology. Edwards *et al.* (5) note that "many ACG areas have yet to be surveyed more than superficially and will contain unrecorded or new species", and during pilot surveys discovered *Agalychnis saltator* at Pitilla, a species new to ACG, absent from all historical lists.

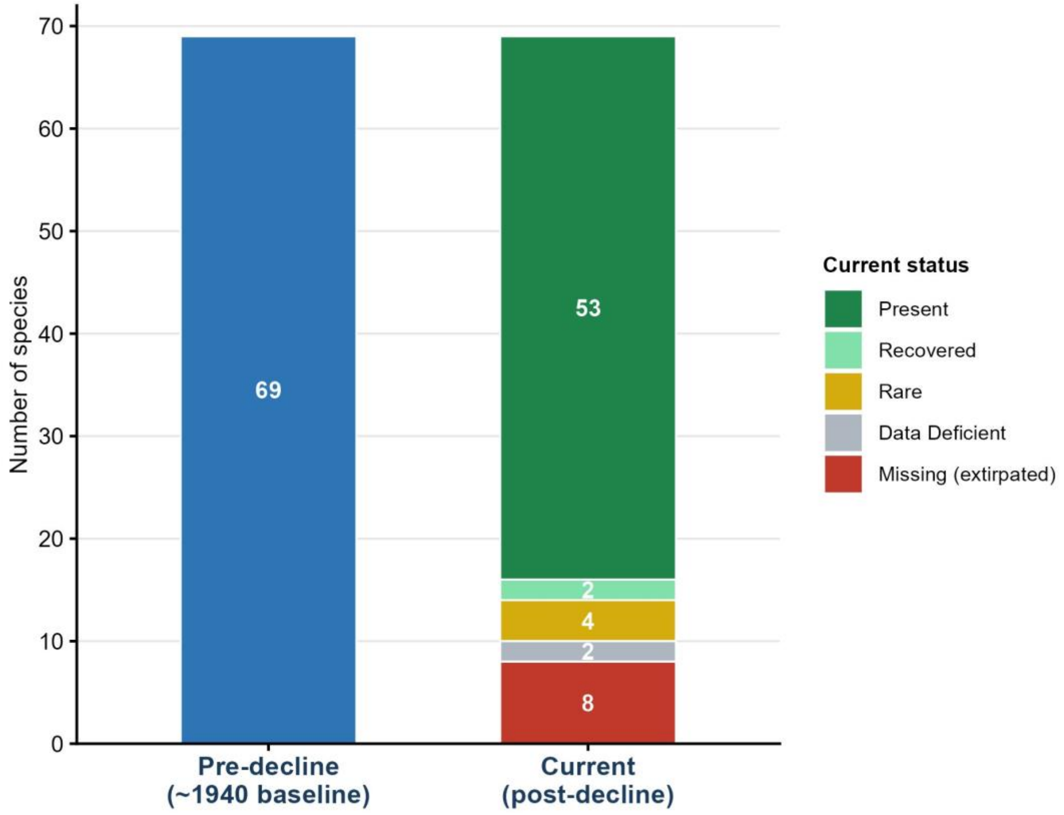

Figure S7. Amphibian species richness in Área de Conservación Guanacaste (ACG), northwestern Costa Rica, before and after the amphibian declines of the 1980s–1990s. The pre-decline baseline (left bar; 69 species) represents the empirically verified minimum fauna reconstructed for approximately 1940 from post-decline field surveys, historical museum collections, and published inventories. It is a conservative floor, not a complete census. Current status categories (right bar) are: Present (n = 53), Recovered (n = 2), Rare (n = 4), Data Deficient (n = 2), and Missing/locally extirpated (n = 8). The 8 Missing species were recorded in ACG before the declines but have not been confirmed in post-decline surveys despite targeted searches. Museum records document a minimum of 80 historical ACG species (Edwards *et al.* 2023), already exceeding the post-decline reconstruction. Taxonomy follows Frost, *Amphibian Species of the World* 6.2 (14).

Estación Cacao: site-level evidence for the scale of decline

Because the major amphibian losses in ACG were concentrated in the highlands, Estación Cacao provides the clearest local expression of the Bd-driven collapse. The comparison with pre-decline data also reveals a deeper limitation of the historical record: some populations disappeared before molecular data could be obtained, meaning that part of the lost diversity was lineage-level rather than only species-level.

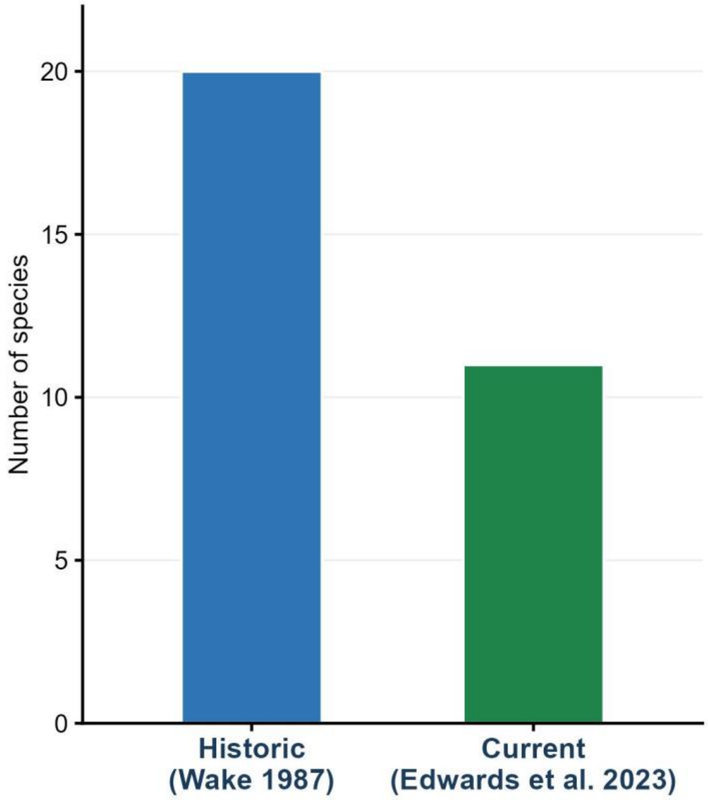

Figure S8. Amphibian species richness at Estación Cacao (Volcán Cacao, ACG cloud forest, ~1,300 m a.s.l.) before and after the amphibian declines of the 1980s–1990s. Historic data (20 species) are from the 1987 UC Berkeley expedition led by D. B. Wake and D. Cannatella. Current data (11 species) are from structured transect surveys (5). These datasets are not directly comparable as standardised community censuses, as the historical collections were made for taxonomic purposes and the recent survey represents approximately one week of fieldwork at this site. The figure documents a 45% reduction in cloud-forest amphibian richness at Cacao over 30 years. The rarefaction curve for the historic Cacao data fails to reach an asymptote (5), (Fig. 2B), confirming that even the 1987 survey was incomplete.

Cryptic diversity among missing ACG amphibians

Two cases directly demonstrate that the loss of named species understates the full erosion of ACG amphibian diversity, because in at least some instances the declines eliminated distinct evolutionary lineages that were never formally recognised.

In *Atelopus*, Costa Rican populations long treated as *A. varius* were not taxonomically uniform: mitochondrial data show that the Monteverde population, historically identified as *A. varius*, represents a possibly undescribed species distinct from populations elsewhere (14). A historical *Atelopus* population was known from the Cacao highlands but disappeared before molecular data could be obtained. Its true taxonomic identity remains unresolved.

The same problem has been demonstrated directly for the rainfrog *Craugastor ranoides*. Sequencing of historical Volcán Cacao specimens collected by Wake and Cannatella, alongside extant dry-forest frogs, showed that what had been treated as a single species contained at least

two mitochondrial lineages in ACG: one grouping with surviving dry-forest populations, and an additional divergent cloud-forest clade of unknown historical distribution (6). This extirpated cloud-forest lineage, possibly an undescribed species, was detectable only because Wake and Canatella preserved tissue samples in ethanol in 1987, an exceptional practice for the era. It is direct empirical proof that morphological surveys compress real species-level diversity, and that the losses in ACG included distinct evolutionary lineages that were never formally recognised.

These cases also illustrate a broader asymmetry between the insect and amphibian datasets underlying this paper. Our analysis rests on the systematic COI barcoding of ACG insects. Amphibians have never been subjected to equivalent systematic molecular sampling anywhere in the world, let alone in ACG. BOLD’s COI reference library for Amphibia is fragmentary because COI is unusually variable in this group and technically difficult to amplify consistently; amphibian taxonomists therefore typically use 16S rRNA, cytochrome b, or multi-marker approaches rather than COI alone. The sequences available in GenBank are overwhelmingly single-species phylogeographic or systematic studies, not community-level surveys of entire faunas across sites. The pre-decline ACG amphibian fauna was assessed using morphology alone. Whatever value is assigned to pre-decline ACG amphibian richness is therefore, by construction, an undercount of unknown magnitude.

Costa Rica's amphibian inventory in a national context

To place the ACG reconstruction in broader perspective, the following figures show that recognized amphibian richness in Costa Rica has continued to rise with no clear asymptote, and that this signal is especially pronounced in salamanders, the group most severely undersampled by pre-molecular surveys and most severely affected by the ACG declines.

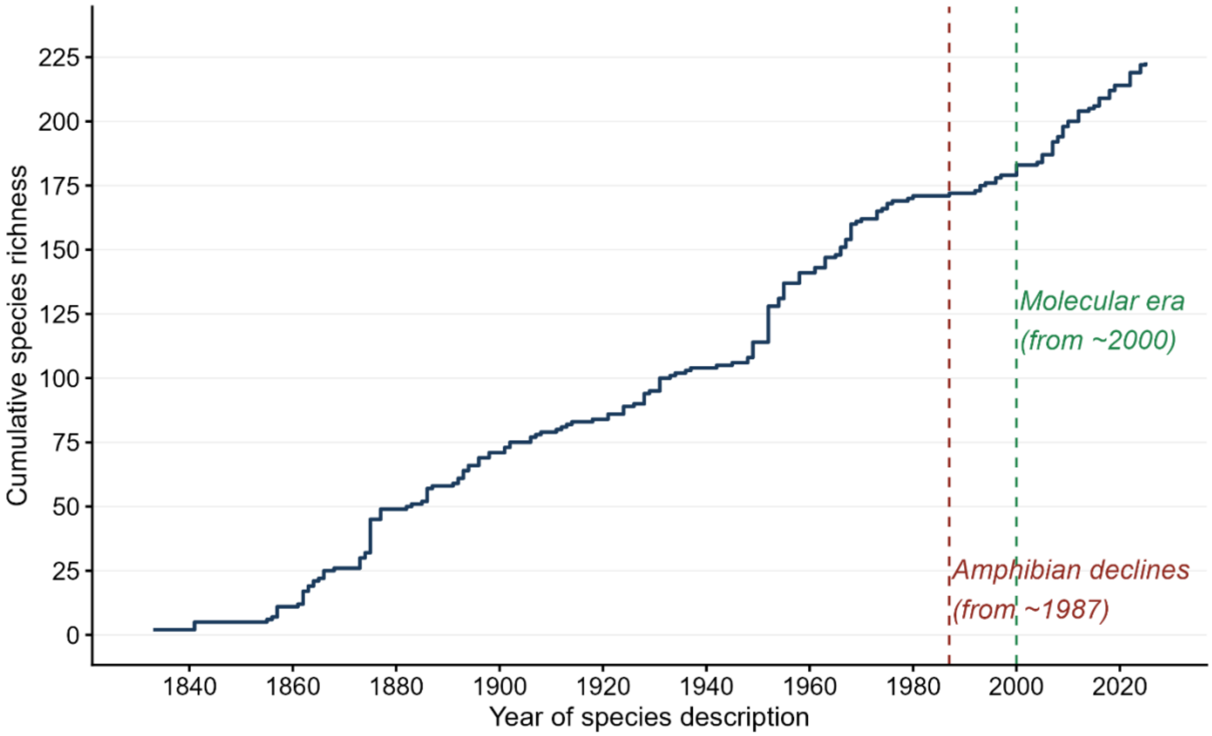

Figure S9 (previous page). Cumulative amphibian species descriptions for Costa Rica, 1835–2025. The figure is based on Frost (13). The dashed red line marks the onset of amphibian declines (~1987); the dashed green line marks the molecular era (~2000). Of 223 currently recognized CR species, 44 (20%) were described only from 2000 or later. The discovery curve has not plateaued. Since ACG encompasses all major Costa Rican ecosystem types, the same incompleteness applies locally: any pre-decline ACG species list is a minimum, not a complete census.

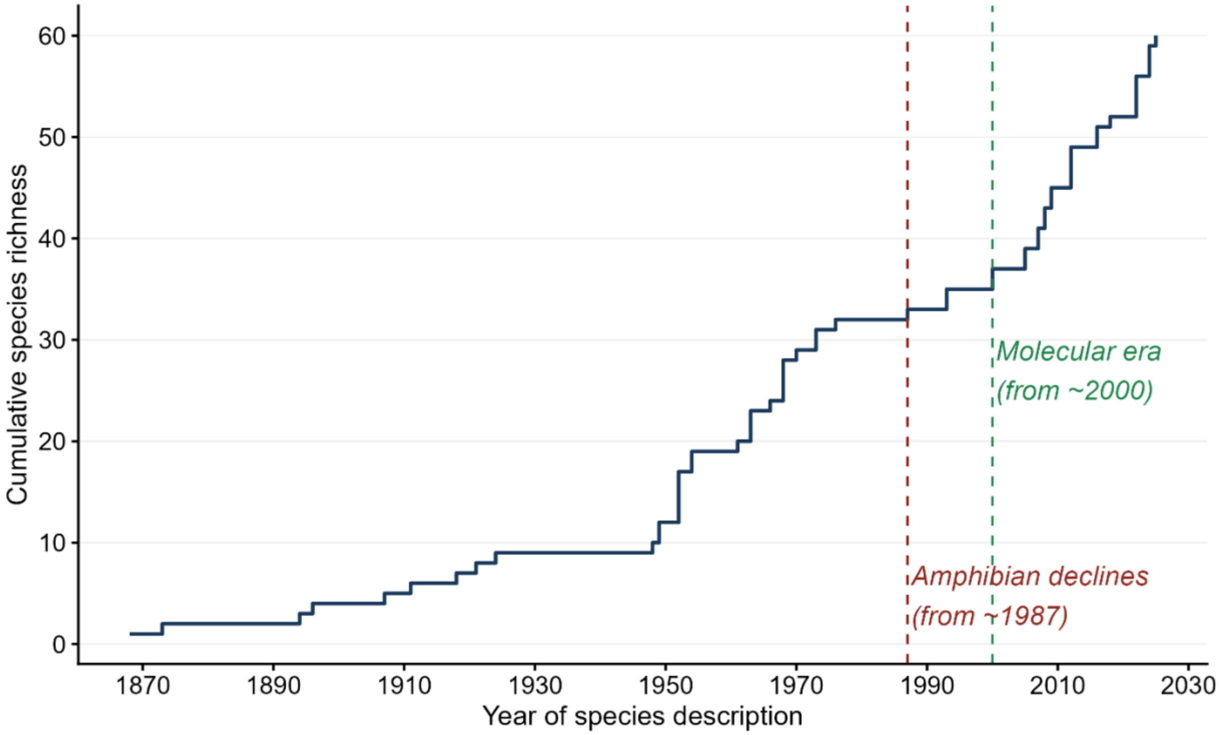

Figure S10. Cumulative salamander (Caudata) species descriptions for Costa Rica. The figure is based on Frost (13). The dashed red line marks the onset of amphibian declines (~1987); the dashed green line marks the molecular era (~2000). Of 60 currently recognized CR salamander species, 25 (42%) were described only from 2000 onward; the discovery curve shows no sign of reaching an asymptote. This post-2000 acceleration reflects improved taxonomic resolution from molecular data, not population recovery. Edwards et al. (5) found zero salamanders across all five ACG survey sites in 2017, despite D.H. Janzen regularly encountering them until the 2000s. Salamander species present in ACG before the Bd collapse but not yet formally described would not appear on any historical species list, even if voucher specimens existed.

Of the five ACG sites surveyed by Edwards *et al.*(5), none reached species richness saturation: Chao1 completeness estimates ranged from 65% at Cacao to 77% at San Gerardo, with three sites below 73% (5) (Table 2). The current detectable fauna is itself substantially undersampled. The pre-decline fauna, assessed with morphology alone, at fewer localities, without molecular tools, was sampled to an even lower degree of completeness.

Conclusion

As with any pre-decline reconstruction, the ACG amphibian estimate for ~1940 carries inherent uncertainty. Three independent lines of evidence—the spatial incompleteness of pre-decline surveys across ACG's 126,000 ha, the 42% of currently recognized Costa Rican salamander species described only since 2000, and the direct demonstration of lineage-level underestimation in *Craugastor ranoides* and *Atelopus varius*, support the conclusion that the true pre-decline richness was substantially higher than the published museum-record baseline of 80 species (5). The estimate of ~160 species applies a 2× correction to that baseline, consistent with the 45% site-level decline documented at Cacao and with the three sources of underestimation described above. As noted in the Rationale, this larger value yields a smaller and more conservative global insect estimate, in keeping with the lower-bound approach of the paper throughout.

SI References

1. R. Cazzolla Gatti *et al.*, The number of tree species on Earth. *Proc. Natl. Acad. Sci. U.S.A.* **119**, e2115329119 (2022).
2. D. Souto-Vilarós *et al.*, Navigating the seven seas of arthropod collection protocols: Metabarcoding arthropod diversity in a tropical forest. *Methods Ecol. and Evol.* **16**, 2395–2407 (2025).
3. A. Chao, R. L. Chazdon, R. K. Colwell, T.-J. Shen, A new statistical approach for assessing compositional similarity based on incidence and abundance data. *Ecol. Lett.* **8**, 148-159 (2005).
4. M. A. Smith *et al.*, "Communities of small terrestrial arthropods change rapidly along a Costa Rican elevation gradient" in *Neotropical Gradients and Their Analysis,* M. RW, Ed. (Springer, 2023), pp. 255-307.
5. A. W. Edwards *et al.*, Amphibian diversity across three adjacent ecosystems in Área de Conservación Guanacaste, Costa Rica. *PeerJ* **11**, e16185 (2023).
6. R. Puschendorf *et al.*, Cryptic diversity and ranavirus infection of a critically endangered Neotropical frog before and after population collapse. *Anim. Conserv.* **22**, 515-524 (2019).
7. K. R. Lips *et al.*, Emerging infectious disease and the loss of biodiversity in a Neotropical amphibian community. *Proc. Natl. Acad. Sci. U.S.A.* **103**, 3165-3170 (2006).
8. J. A. Pounds, M. L. Crump, Amphibian declines and climate disturbance: The case of the Golden Toad and the Harlequin Frog. *Conserv. Biol.* **8**, 72-85 (1994).
9. J. A. Pounds, M. P. L. Fogden, J. H. Campbell, Biological response to climate change on a tropical mountain. *Nature* **398**, 611-615 (1999).
10. J. A. Pounds, M. P. L. Fogden, J. M. Savage, G. C. Gorman, Tests of Null Models for Amphibian Declines on a Tropical Mountain. *Conserv. Biol.* **11**, 1307-1322 (1997).
11. B. E. Young *et al.*, Population Declines and Priorities for Amphibian Conservation in Latin America. *Conserv. Biol.* **15**, 1213-1223 (2001).

12. J. Abarca, G. Chaves, A. Garcia-Rodriguez, R. Vargas, Reconsidering extinction: rediscovery of Incilius holdridgei (Anura: Bufonidae) in Costa Rica after 25 years. *Herpetol. Rev.* **41**, 150-152 (2010).
13. D. Frost, Amphibian Species of the World: an Online Reference. American Museum of Natural History, New York. Deposited April 2026.
14. J. Ramírez, C. Jaramillo, E. Lindquist, A. Crawford, R. Ibáñez, Recent and Rapid Radiation of the highly endangered harlequin frogs (*Atelopus*) into Central America inferred from mitochondrial DNA sequences. *Diversity* **12**, 360 (2020).
